# Normal levels of KIF5 but reduced KLC1 levels in both Alzheimer disease and Alzheimer disease in Down syndrome: evidence suggesting defects in anterograde transport

**DOI:** 10.1186/s13195-021-00796-6

**Published:** 2021-03-10

**Authors:** Xu-Qiao Chen, Utpal Das, Gooho Park, William C. Mobley

**Affiliations:** Department of Neurosciences, University of California San Diego, La Jolla, CA 92093 USA

**Keywords:** Alzheimer’s disease, Down syndrome, Axonal transport, KIF5, APP, Dp16, KLC1

## Abstract

**Background:**

Impaired axonal transport may contribute to the pathogenesis of neurodegenerative diseases, including Alzheimer’s disease (AD) and Down syndrome (DS). Axonal transport is a complex process in which specific motor proteins move cargoes to and from neuronal cell bodies and their processes. Inconsistent reports point to the changes in AD in the levels of the classical anterograde motor protein kinesin family member 5 (KIF5) and the primary neuronal KIF regulator kinesin light chain 1 (KLC1), raising the possibility that anterograde transport is compromised in AD.

**Methods and materials:**

To address inconsistencies and determine if the shared pathologies in AD and elderly DS subjects with dementia (AD in DS; AD-DS) extend to the changes in KIF5 and KLC1, we measured the levels of all the three KIF5 family members and KLC1 in the AD and AD-DS frontal cortex and AD temporal cortex and cerebellum in samples taken with a short postmortem interval. To support future studies to explore the cell biological basis for any changes detected, we also examined the levels of these proteins in the brains of young and aged adult mice in the Dp (16)1Yey/+ (Dp16) mouse model of DS and J20 mouse model of AD.

**Results:**

There were no changes in comparison with controls in KIF5 family members in either the AD or AD-DS samples when normalized to either β-actin or glyceraldehyde-3-phosphate dehydrogenase (GAPDH). Interestingly, however, samples from control brains as well as from AD and AD-DS demonstrated strong positive correlations between the levels of KIF5 family members, suggesting positive co-regulated expression. Importantly, while earlier reports pointed to a negative correlation between the levels of the amyloid precursor protein (APP) and KIF5A levels, we found the opposite to be true in AD-DS; this was especially striking given triplication of the APP gene, with increased APP protein levels. AD and control samples showed positive correlations between fl-hAPP and KIF5 members, but they were less consistent. In contrast to the findings for KIF5, the levels of KLC1 were downregulated in the frontal cortex of both AD and AD-DS brains; interestingly, this change was not seen in the AD temporal cortex or cerebellum. As postmortem interval has a negative effect on the levels of KLC1, but not KIF5 members, we analyzed a subset of samples with a very short postmortem interval (PMI) (≤ 6 h), a PMI that was not significantly correlated with the levels of KLC1 in either AD or AD-DS samples; we confirmed the presence of a statistically significant reduction of KLC1 in AD and AD-DS brains as compared with control brains. Studies comparing Dp16 to its euploid control recapitulated human studies in demonstrating no change in KIF5 levels and a positive correlation between the levels of KIF5 family members. J20 mice also showed normal KIF5 levels. However, unlike the AD and AD-DS frontal cortex, KLC1 levels were not reduced in the brains of Dp16 or J20 mice.

**Conclusion:**

These data point to significant reductions in KLC1 in AD and AD-DS. In so doing, they raise the possibility of compromised KLC1-mediated axonal transport in these conditions, a posit that can now be pursued in model systems in which KLC1 expression is reduced.

**Supplementary Information:**

The online version contains supplementary material available at 10.1186/s13195-021-00796-6.

## Background

The unique, polarized morphology of neurons with their long axon and multiple shorter dendrites enforces the need for accurate and efficient cargo transport to support the structure and function of both neuronal somas and their processes. Cargoes are thus moved both anterogradely and retrogradely in axons and dendrites. Anterograde cargoes are diverse and include proteins, lipids, ribonucleic acids (RNAs), and organelles; retrograde transport also engages diverse cargoes to inform somas. Among the latter are neurotrophic signals [1, 2]. Axonal transport is mediated by motor protein complexes [3]. Kinesin family members are responsible for anterograde axonal transport; dynein and some kinesin members, including the KIFC subfamily, mediate retrograde axonal transport [3, 4]. Axonal transport defects have been linked to multiple neurodegenerative diseases [1, 2]. Deficits in both anterograde [5–7] and retrograde axonal transport [8–12] have been extensively studied in neurological disorders including Alzheimer’s disease (AD), Down syndrome (DS), Parkinson disease (PD), Huntington disease (HD), and amyotrophic lateral sclerosis (ALS). The findings have encouraged the view that axonal transport is a potential target for the development of therapeutics [1, 13].

AD is the most common cause of dementia, accounting for up to 70% of cases [14]. Clinical manifestations include memory loss, cognitive decline and behavioral dysfunction, and failure to maintain activities of daily living [15]. AD constitutes a leading cause of death with survival from diagnosis averaging 8 years for those diagnosed at age 65 [16]. The neuropathology of AD manifests in several features including amyloid plaques and neurofibrillary tangles (NFTs) [17].

DS, also known as trisomy 21, is due to trisomy for all or part of human chromosome 21 (HSA21). DS is usually associated with growth delay, intellectual disability, and distinctive facial features. In addition, those with DS have a markedly increased risk for AD [18–20]. Indeed, essentially, all adults with DS demonstrate AD-like pathology by 40 years of age, including amyloid plaques and NFTs, with dementia onset in most by age 60 [17–19], leading to the designation of this condition as AD in DS (AD-DS). The data are compelling that increased *APP* gene dose is necessary for AD in DS [21–23] but how increased APP levels act to advance pathogenesis remains an active area of interest. Aberrant axonal transport represents one focus, and changes in axonal transport are supported in studies in models of AD and AD-DS [1, 12, 24–27]. Indeed, the evidence suggests that changes in axonal transport may significantly predate loss of synapses, axonal dysfunction, and neurodegeneration [26]. As yet unclear, however, is what role(s) in pathogenesis may be played by deficits in anterograde and retrograde transport.

Kinesin isoforms play a key role in anterograde cargo transport. Among kinesin family members, conventional Kinesin-1 is a heterotetramer consisting of two kinesin heavy chains [KHCs (kinesin family member 5 s, KIF5s)] and two kinesin light chains (KLCs). The KHCs employ ATPase activity to generate anterograde motility; in turn, KLCs (KLC1 and KLC2) support the attachment of cargoes to the Kinesin-1 complex [4]. The KIF5 family, comprising isoforms KIF5A, KIF5B, and KIF5C, is known to mediate the anterograde delivery of APP and its processing enzymes as well as many other cargoes, including messenger ribonucleic acid (mRNA)-protein complexes, mitochondria, and neurotrophins and their receptors [4, 28–31]. KIF5A and KIF5C are exclusively expressed in neurons; in contrast, KIF5B is ubiquitously expressed. KLC1 is enriched in neurons; KLC2 is ubiquitously distributed [32, 33]. The significance of the roles played by KIF5s and KLC1 in axonal transport is supported by the phenotypes demonstrated in mice in which these genes have been deleted, including loss of motor neurons, loss of axons, motor abnormalities, abnormal accumulation of cargoes, and reduced brain size [4].

The gene for APP encodes a full-length APP protein (fl-APP) and its products, including transmembrane C-terminal fragments (CTFs) as well as the Aβ peptides that accumulate in amyloid plaques and whose presence in toxic oligomeric species is viewed as an essential contributor to pathogenesis [17, 34]. fl-APP is processed by sequential endopeptidase cleavages. APP internalization and transport contribute to processing [35, 36]. In turn, APP products have been shown to impact axonal transport [8, 11, 37–40]. The 99 amino-acid CTF (C99) was shown to impact retrograde transport of endosomal cargoes [11, 41]; the evidence points to increased levels of C99 as also reducing anterograde transport of APP [38]. In addition, in Aβ-treated hippocampal neurons, anterograde axonal transport of some cargoes, like mitochondria, was more affected than retrograde transport [40]. While the underlying mechanism is yet to be fully defined, these data raise the possibility that APP and its products impact axonal transport. Recent studies have addressed one possibility that is changes of motor proteins, but with conflicting findings. In one study of AD brain, there was an increased KIF5A mRNA level with a very small increase in the protein [42]. The finding for KIF5A protein was later challenged by a study showing marked downregulation of both KIF5A and KIF5B protein in AD [43]. Reduced levels of KIF5A and KIF5B were also detected in the 5XFAD mouse cortex and the level of KIF5A in cultures of wild-type mouse brain neurons was reduced after treating with 1 μM oligomeric Aβ1–42 [43]. KIF5C levels were also reported to be reduced in familial, but not sporadic, AD [44]. Somewhat more consistent findings have been reported for changes in KLC1 in AD, with decreased protein levels [45–47] speculated to contribute to reduced anterograde axonal transport; nevertheless, others found no change in KLC1 [48]. In summary, existing observations with respect to AD are inconsistent, thus preventing a conclusion with respect to whether or not changes in KIF5 family members and KLC1 could contribute to changes in anterograde transport.

Shared neuropathological findings in AD and AD-DS motivated us to explore possible common differences relative to normal controls with respect to proteins that support anterograde transport. We examined KIF5 members and KLC1 in short postmortem interval (PMI) AD and AD-DS brains and normalized to both β-actin and glyceraldehyde-3-phosphate dehydrogenase (GAPDH). We found no change in the levels of KIF5 proteins in AD and AD-DS as compared to controls. Interestingly, the levels of KIF5 family members were highly correlated with each other when examining AD, AD-DS, and controls. Moreover, positive correlations between all KIF5 members and fl-hAPP levels were consistently detected in the AD-DS group; positive correlations in AD were seen with all KIF5 members and fl-hAPP when normalizing to GAPDH and in controls only when normalized with β-actin. We found that KLC1 levels were significantly reduced in the frontal cortex of both AD and AD-DS brains. The reduction in KLC1 was not seen in the AD temporal cortex and cerebellum. To support future studies to explore the cell biological basis for any changes detected, we also examined the levels of these proteins in the brains of a well-established model of DS, the Dp16 mouse. The Dp16 mouse provides an optimal genetic model for DS in that it harbors three copies of the ~ 115 mouse chromosome 16 genes homologous to human genes present on the long arm of chromosome 21 [49]. These mice have three copies of *APP* and demonstrate features of AD pathology [50, 51]. Like AD and AD-DS samples, the Dp16 brain showed no change in KIF5 levels nor were changes recorded in the J20 mouse model of AD. However, unlike AD and AD-DS frontal cortex, KLC1 levels were not reduced in the Dp16 or J20 brain. Taken together, these findings point to reductions in KLC1, but not KIF5 family members, in the frontal cortex of both AD and AD-DS and raise the possibility that this change results in compromised anterograde transport in these disorders.

## Materials and methods

### Animals

Dp (16)1Yey/+ (Dp16) mice were obtained from the Jackson Laboratory (stock #: 013530) and maintained by crossing females to male (C57BL/6J × C3H/HeJ) F1 mice (B6C3). Diploid (2N) littermate mice on the same background served as controls. The genotype of all animals was confirmed by polymerase chain reaction (PCR). For genotyping, tail samples were used to extract genomic DNA. A protocol was used to amplify the HPRT insertion which is only found in Dp16 mice along with amplification of the IL-2 gene as an internal control. The primer sequences used for HPRT were fwd: 5′-AGGATGTGATACGTGGAAGA, rev: 5′-CCAGTTTCACTAATGACACA, while the primers for IL-2 were fwd: 5′-CTAGGCCACAGAATTGAAAGATCT, rev: 5′-GTAGGTGGAAATTCTAGCATCATCC.

J20 mice were purchased from the Jackson Laboratory (B6.Cg-*Zbtb20*^*Tg(PDGFB-APPSwInd)20Lms*^/2Mmjax; Stock No: 34836-JAX) and were carried on the C57BL/6J background. APP knockout mice [52] which have been carried out on the C57BL/6J background for more than ten generations were also obtained from the Jackson Laboratory (B6.129S7-*App*^*tm1Dbo*^/J; Stock No: 004133). Genotyping of all animals was performed following the PCR-based protocol provided by the Jackson Laboratory. Wild-type mice on the C57BL/6J background served as controls for both J20 and APP knockout mice.

All animals were maintained and bred according to standard procedures. Mice were housed 2 to 5 per cage with a 12-h light-dark cycle and ad lib access to food and water. Male mice were taken for all studies of KIF5 and KLC1 levels using sample sizes sufficient to detect statistically significant differences of ≥20%. Sacrifice was between 7 AM and 7 PM daily; best efforts were made to minimize suffering.

### Plasmids and antibodies

Rat KIF5A cDNA was cloned into pEGFP-N3 (Clontech) to create construct KIF5A-EGFP. Similarly, mouse KIF5B and KLC1 cDNAs were cloned into the pEGFP-C vector to obtain EGFP-KIF5B and EGFP-KLC1, respectively. pGFP-KIF5C was from Addgene (#71853). Rabbit antibodies anti-KIF5A (1:2000; 21186-1-AP), KIF5B (1:2000; 21632-1-AP), KIF5C (1:2000; 25897-1-AP), and mouse antibody anti-β-actin (1:10,000; 60008-1-Ig) were obtained from Proteintech (Rosemont, IL); rabbit anti-APP antibody (1:5000; A8717; St. Louis, MO) was purchased from MilliporeSigma; mouse anti-GFP antibody (1:2000; sc-9996) and goat anti-KLC1 antibody (1:1000; sc-13362) were from Santa Cruz Biotechnology (Dallas, TX). The mouse anti-GAPDH antibody (1:3000; GTX627408) was from GeneTex (Irvine, CA).

### Cell culture and transfection

HEK293T cells were from the American Type Culture Collection (ATCC-CRL-3216; ATCC, MD) and were cultured in DMEM (10-013-CV; Corning; Corning, NY) containing 10% fetal bovine serum (FB-02; Omega Scientific; Tarzana, CA) and 1% penicillin-streptomycin (15140122; Thermo Fisher Scientific; Waltham, MA). Transient expression was performed with polyethylenimine (408,727; MilliporeSigma) according to the manufacturer’s protocol; fresh medium was supplied 4–6 h post-transfection. The expression of constructs was evaluated 36 h after transfection by Western blotting.

### Human sample processing

Human AD, AD-DS, and control frontal cortexes (male and female) (AD, *n* = 12, age 64–93; and control for AD (C/AD), *n* = 12, age 48–95 (Table 1); AD-DS, *n* = 21, age 41–65; and control for AD-DS (C/AD-DS), *n* = 19, age 39–95 (Table 2)) and human AD temporal cortex and cerebellum (AD, *n* = 7, age 64–93 and control for AD (C/AD), *n* = 8, age 58–95 (Table 3)) were obtained from Banner Sun Health Research Institute, UCI MIND, and NIH NeuroBioBank. The AD dementia status of those with DS (AD-DS) was listed as confirmed in the samples provided by the NIH. In the cases from UCI, the diagnosis of AD-DS followed comprehensive assessments including medical history, neurological examination, and neuropsychological evaluation with particular attention given to other health co-morbidities or neuropsychiatric conditions that might cause or mimic dementia. The diagnosis of AD-DS was based upon a consensus opinion from two or more independent raters after a comprehensive evaluation of existing records and data. Participants were classified as demented if there was a history of progressive memory loss, disorientation, and functional decline over a period of at least 6 months. Samples of approximately 10 to 20 mg frozen tissue were processed in 0.5 ml RIPA buffer (50 mM Tris-HCl pH 8.0, 150 mM NaCl, 1% NP-40, 1% Triton X-100, 0.1% sodium deoxycholate, 0.1% sodium dodecyl sulfate and protease inhibitor cocktail [4693124001; MilliporeSigma]) and rotated in 4 °C for 30 min. All lysates were centrifuged (12,000 rpm for 15 min at 4 °C) to produce supernatants, and the protein content of supernatants was determined using the Bradford assay (5000001, Bio-Rad, Hercules, CA). Samples were examined using Western blotting analysis.

**Table 1:** Demographics, PMI, and clinical diagnosis for AD and C/AD frontal cortex samples

Patient	Gender	Age (years)	Post-mortem interval (h)	Diagnosis
C/AD 1	Male	79	3	Cognitively normal
C/AD 2	Male	80	3.5	Cognitively normal
C/AD 3	Male	93	3	Cognitively normal
C/AD 4	Female	58	3.1	Cognitively normal
C/AD 5	Female	59	3.1	Cognitively normal
C/AD 6	Female	95	2.5	Cognitively normal
C/AD 7	Female	49	7	Cognitively normal
C/AD 8	Female	54	6	Cognitively normal
C/AD 9	Female	59	5.9	Cognitively normal
C/AD 10	Male	48	6	Cognitively normal
C/AD 11	Male	57	16	Cognitively normal
C/AD 12	Male	58	9	Cognitively normal
Mean ± SEM	6F/6M	66 ± 5	5.7 ± 1.1	
AD 1	Male	64	2.3	AD
AD 2	Male	74	2.6	AD
AD 3	Male	82	3	Mixed vascular dementia
AD 4	Male	89	2.2	AD
AD 5	Female	84	2.5	AD
AD 6	Female	89	2.7	AD
AD 7	Female	93	2.8	AD
AD 8	Female	73	18	AD
AD 9	Female	70	12	AD
AD 10	Female	77	15	AD
AD 11	Female	80	7	AD
AD 12	Male	78	23	AD
Mean ± SEM	7F/5M	79 ± 3	7.8 ± 2.1

**Table 2:** Demographics, PMI, and clinical diagnosis for AD-DS and C/AD-DS frontal cortex samples

Patient	Gender	Age (years)	Post-mortem interval (h)	Diagnosis
C/AD-DS 1*	Female	95	2.5	Cognitively normal
C/AD-DS 2*	Male	93	3	Cognitively normal
C/AD-DS 3*	Male	79	3	Cognitively normal
C/AD-DS 4	Male	74	3.3	Cognitively normal
C/AD-DS 5*	Female	58	3.1	Cognitively normal
C/AD-DS 6*	Female	49	7	Cognitively normal
C/AD-DS 7*	Female	54	6	Cognitively normal
C/AD-DS 8*	Female	59	5.9	Cognitively normal
C/AD-DS 9*	Male	48	6	Cognitively normal
C/AD-DS 10*	Male	57	16	Cognitively normal
C/AD-DS 11*	Male	58	9	Cognitively normal
C/AD-DS 12	Female	50	7	Cognitively normal
C/AD-DS 13	Female	46	18	Cognitively normal
C/AD-DS 14	Female	39	19	Cognitively normal
C/AD-DS 15	Male	59	5.9	Cognitively normal
C/AD-DS 16	Male	59	15.8	Cognitively normal
C/AD-DS 17	Female	48	8	Cognitively normal
C/AD-DS 18	Female	51	21	Cognitively normal
C/AD-DS 19	Female	57	7	Cognitively normal
Mean ± SEM	11F/8M	60 ± 3	8.8 ± 1.4	
AD-DS 1	Female	45	2.7	Trisomy 21 with AD
AD-DS 2	Male	46	6.4	Trisomy 21 with AD
AD-DS 3	Male	49	2.2	Trisomy 21 with AD
AD-DS 4	Male	55	4.5	Trisomy 21 with AD
AD-DS 5	Female	57	6	Trisomy 21 with AD
AD-DS 6	Female	46	7	Trisomy 21 with AD
AD-DS 7	Male	65	10	Trisomy 21 with AD
AD-DS 8	Male	56	16	Trisomy 21 with AD
AD-DS 9	Male	55	12	Trisomy 21 with AD
AD-DS 10	Male	57	5	Trisomy 21 with AD
AD-DS 11	Female	51	4	Trisomy 21 with AD
AD-DS 12	Male	57	3	Trisomy 21 with AD
AD-DS 13	Male	41	15	Trisomy 21 with AD
AD-DS 14	Male	64	20	Trisomy 21 with AD
AD-DS 15	Male	51	15.7	Trisomy 21 with AD
AD-DS 16	Male	50	22.8	Trisomy 21 with AD
AD-DS 17	Female	45	2.75	Trisomy 21 with AD
AD-DS 18	Female	47	6.5	Trisomy 21 with AD
AD-DS 19	Female	50	5	Trisomy 21 with AD
AD-DS 20	Female	52	4.37	Trisomy 21 with AD
AD-DS 21	Female	62	2.42	Trisomy 21 with AD
Mean ± SEM	9F/12M	52 ± 1	8.3 ± 1.4	

*The control samples also listed as C/AD in Table 1; these samples together with the remaining controls constituted the samples designated as C/COMB

**Table 3:** Demographics, PMI, and clinical diagnosis for AD and C/AD temporal cortex and cerebellum samples

Patient	Gender	Age (years)	Post-mortem interval (h)	Diagnosis
C/AD 1	Male	74	3.3	Cognitively normal
C/AD 2	Male	74	4.6	Cognitively normal
C/AD 3	Male	79	3	Cognitively normal
C/AD 4	Male	80	3.5	Cognitively normal
C/AD 5	Male	93	3	Cognitively normal
C/AD 6	Female	58	3.1	Cognitively normal
C/AD 7	Female	59	3.1	Cognitively normal
C/AD 8	Female	95	2.5	Cognitively normal
Mean ± SEM	3F/5M	77 ± 5	3.3 ± 0.2	
AD 1	Male	64	2.3	AD
AD 2	Male	74	2.6	AD
AD 3	Male	82	3	Mixed vascular dementia
AD 4	Male	89	2.2	AD
AD 5	Female	84	2.5	AD
AD 6	Female	89	2.7	AD
AD 7	Female	93	2.8	AD
Mean ± SEM	3F/4M	82 ± 4	2.6 ± 0.1	

### Mouse brain dissection and homogenization

Brains were dissected using established landmarks. For Dp16 and age-matched 2N mice, the forebrain was isolated by first removing the olfactory bulbs; cerebellum and brain stem were then removed via a cut anterior to the cerebellum and extending through the posterior midbrain. Dissected forebrain was then immediately frozen in dry ice. The whole brains of J20 and *APP* knockout mice and age-matched wild-type mice, minus olfactory bulbs, were also dissected and frozen in dry ice. The brain, kidney, and liver were dissected from 2N adult mice to evaluate antibody specificity. Brain tissues were homogenized via a Dounce homogenizer in RIPA buffer (50 mM Tris-HCl pH 8.0, 150 mM NaCl, 1% NP-40, 0.5% sodium deoxycholate, 0.1% sodium dodecyl sulfate, and protease inhibitor cocktail) (1 ml for each hemibrain) and rotated at 4 °C for 30 min. All lysates were centrifuged (12,000 rpm for 15 min at 4 °C) to produce supernatants, and the protein content of supernatants was measured by Bradford assay.

### SDS-PAGE immunoblotting

Established protocols were followed for SDS-PAGE/immunoblotting [53]. Equal amounts of total proteins for each sample (10–20 μg) were separated on SDS-PAGE and then electrotransferred to nitrocellulose membranes (Bio-Rad). The membranes were blocked with 5% nonfat milk for 1 h and probed with specific primary antibodies overnight at 4 °C followed by incubation with goat anti-rabbit IgG-HRP (1:15,000; 111-035-144) or anti-mouse IgG–HRP (1:15,000; 115-035-003) or donkey anti-goat IgG-HRP (1:15,000; 705-035-003) (Jackson ImmunoResearch Laboratories; West Grove, PA) at room temperature for 1 h. To control for false-positive signals, we used rabbit normal IgG (011-000-003, Jackson ImmunoResearch Laboratories) and an unrelated goat IgG (cathepsin D, sc-6486, Santa Cruz Biotechnology) at comparable working concentrations. All blots were developed using the BioRad Clarity Western ECL substrate and captured using ChemiDoc XRS + (Bio-Rad); only blots within signals in the linear range were quantitated using the ImageLab 3.0.1 software (Bio-Rad).

### mRNA measurements

Total RNA was extracted from AD-DS frontal cortexes obtained from NIH NeuroBioBank using Quick RNA Miniprep Kit (Zymo Research, USA) and equal amounts of total RNA were used for cDNA generation with iScript™ cDNA Synthesis Kit (Bio-Rad) following the manufacturer’s instructions [8]. The primer sequences used for KIF5A were fwd: 5′-TTACCTGGACAAAATTCGTGACC, rev: 5′-GGTGACAGCCACATGACGAT; KIF5B: fwd: AGATCCTGCGGAACACTATTCA, rev: 5′-GCGGTTGCTGGTTTATCATTGG, while the primers for KIF5C were fwd: 5′-ATGTCTTCGACAGAGTGCTACC, rev: 5′-ACGCAAAAATCGTCCCGTTAT; KLC1: fwd: GTGAGGCACAGGTTATGATGG, rev: GTTCATCCCGTAGCCACTGAT, all from PrimerBank [54]. Polymerase chain reaction was for 40 cycles. Endogenous GAPDH mRNA was used as the internal control with the sequences: fwd: 5′-GCCACATCGCTCAGACACC, rev: 5′-AATCCGTTGACTCCGACCTTC. Values within the log-linear phase of the amplification curve were defined for each probe/primers set and analyzed using the ΔΔCt method (Applied Biosystems 7300 Real-Time PCR System).

### Statistics

All data are presented as the mean ± SEM. Statistical analyses were performed using PRISM (GraphPad Software Inc., La Jolla, CA) with a two-tailed Mann-Whitney test. Spearman’s correlation was used to assess a relationship between proteins. The significance levels were *P < 0.05, **P < 0.01, and ***P < 0.001 unless stated, n.s., non-significant. Controls for the AD samples (C/AD) and for the AD-DS samples (C/AD-DS) extensively overlapped in age; for some analyses, they were examined as a group (C/COMB).

## Results

### Antibodies against the KIF5 family members detected proteins of the predicted size

**Fig. 1:**
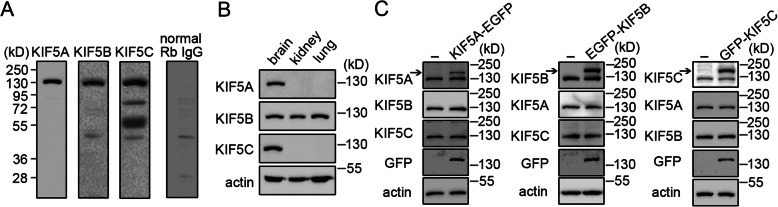
Validating antibodies used for KIF5 family members. **a** Western blotting was performed using the lysates of 2N adult mouse brain. Predicted molecular weights of mouse KIF5A, KIF5B, and KIF5C are 117, 110, and 109 kDa, respectively. Using normal rabbit IgG, we failed to detect specific signals at these molecular weights. **b** Lysates were prepared from the brain, kidney, and liver of 2N adult mice to compare the levels of KIF5 in tissues by Western blotting. **c** A plasmid encoding KIF5A-EGFP, EGFP-KIF5B, or GFP-KIF5C was transiently expressed in HEK293T cells for 36 h followed by cell lysis and Western blotting to evaluate the specificity of KIF5 antibodies. GFP was probed to indicate the presence of KIF5A-EGFP, EGFP-KIF5B, or GFP-KIF5C. β-Actin was employed as an internal control. Arrow indicates transfected KIF5s

To establish the validity and utility of the antibodies used herein, antibodies were first subjected to immunoblotting with brain lysates from the wild-type adult mouse (Fig. 1a). Each antibody revealed a distinct band of approximately the calculated molecular weight (KIF5A, 117 kDa; KIF5B, 110 kDa; KIF5C, 109 kDa). No band at the expected molecular weight was detected when the same mouse brain lysates were probed with normal IgG from rabbits (Fig. 1a). The anti-KIF5A antibody recognized a band slightly larger than that detected by anti-KIF5B and anti-KIF5C antibodies; the anti-KIF5B and anti-KIF5C antibodies recognized bands of about the same molecular weight (Fig. 1a) [32]. Other bands were observed, possibly reflecting non-specific binding or alternative splicing. KIF5A and KIF5C are highly expressed in the brain, as compared with other tissues, while KIF5B is ubiquitously expressed [32]. As a further test for the antibodies, we prepared lysates from the kidney and liver of wild-type adult mice and compared the levels of KIF5 in these tissues. As predicted, both KIF5A and KIF5C antibodies detected a band only in brain samples, while KIF5B detected a band with comparable intensities in all tissues (Fig. 1b). Further evidence for antibody specificity was that in HEK293T cells, only the KIF5A antibody recognized the transfected KIF5A-EGFP, only the KIF5B antibody detected the transfected EGFP-KIF5B, and only the KIF5C antibody detected the transfected pGFP-KIF5C (Fig. 1c). These data support the use of these KIF5 antibodies to detect KIF5 members by Western blotting.

### Neither the levels of KIF5 family members nor APP were changed in the AD brain

**Fig. 2:**
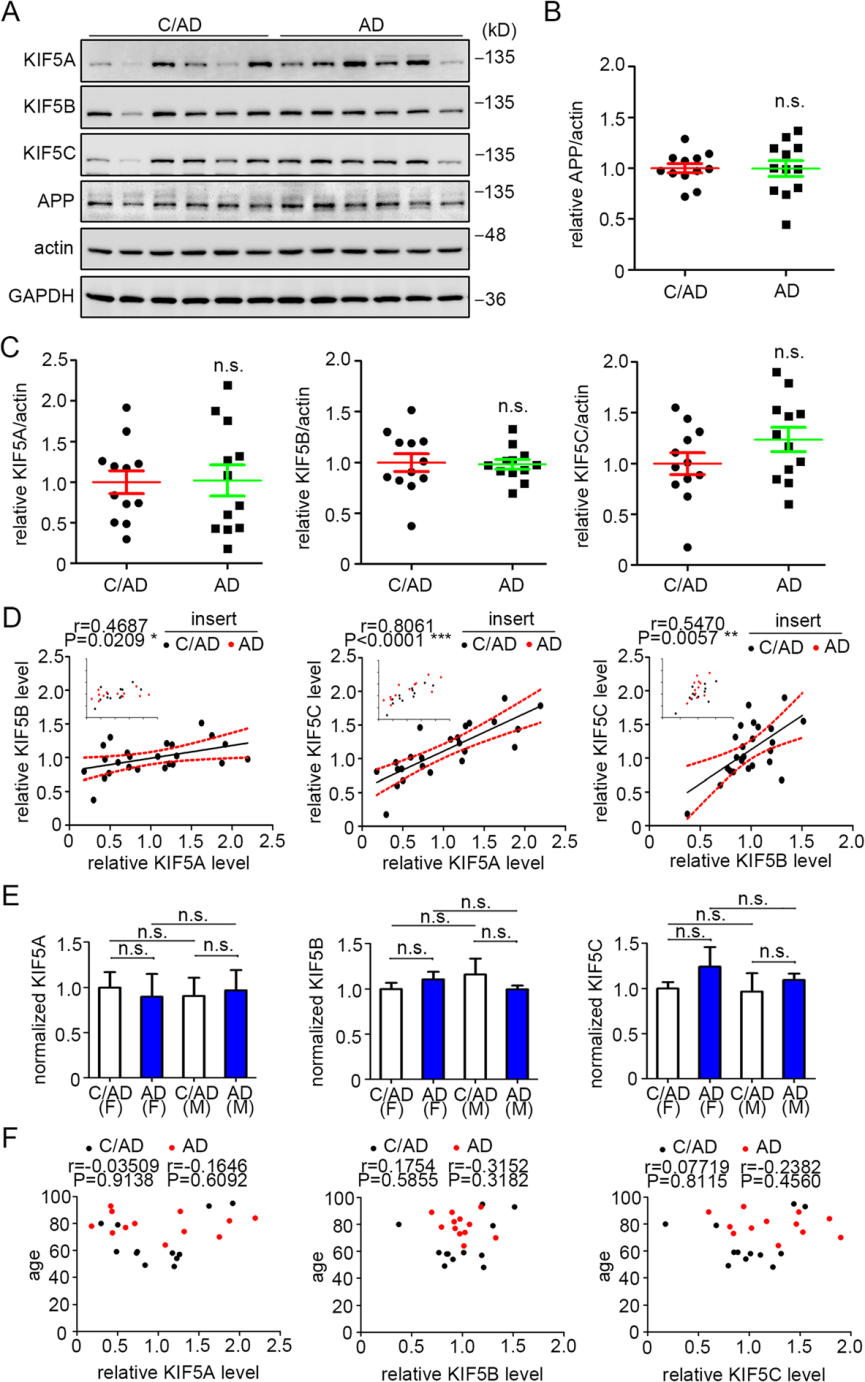
Neither the levels of KIF5 family members nor APP were changed in the frontal cortex of AD brain. **a** Western blotting of KIF5 levels in protein extracts from the frontal cortex of patients with AD and C/AD. fl-hAPP was also probed. β-Actin and GAPDH were each used as loading controls. **b** Quantitation and statistical analysis of the levels of fl-hAPP in AD and C/AD. **c** Quantitation and statistical analysis of the levels of KIF5 family members in AD and C/AD. **d** Spearman’s correlation analysis of KIF5 members in combined C/AD and AD samples; the red dotted lines mark the 95% confidence interval. Insert with the same diagram indicates the distribution of C/AD and AD samples. **e** Quantitation and statistical analysis of the levels of KIF5 family members in females and males from AD and C/AD samples. **f** Spearman’s correlation analyses for age and the levels of KIF5 members in AD and C/AD. *n* = 12 for both C/AD and AD patients; Mann-Whitney test in **b**, **c**, and **e**. n.s., non-significant; F, female; M, male

Changes in KIF5A expression have been linked to AD, but with inconsistent findings [42, 43, 48]. To further examine this question, we examined the levels of KIF5 family members in postmortem AD brains and in age- and PMI-comparable controls (Table 1). All AD subjects were at Braak stages V–VI. To capture one brain region impacted by AD pathology, proteins were extracted from the frontal cortex, and the levels of KIF5 family members and full-length human APP (fl-hAPP) were evaluated after normalization to endogenous β-actin. As expected, fl-hAPP in AD samples was comparable to that in age-matched controls (i.e., C/AD) (Fig. 2a, b). While the levels for each of the KIF5 members varied across C/AD and AD samples, the average level of each of the three KIF5 family members was no different in AD from C/AD (Fig. 2a, c). Interestingly, the levels of KIF5 family members were strongly correlated with one another when the values for the AD and C/AD cases were combined, suggesting co-regulation (Fig. 2d). Separating AD and C/AD, a comparable positive correlation of KIF5 members was seen except between KIF5A and KIF5B in AD and between KIF5B and KIF5C in C/AD (Table 4). Finally, neither gender nor age had a demonstrable effect on the levels of KIF5 family members (Fig. 2e, f).

**Table 4:** Spearman’s correlation analysis between KIF5 members when normalized to β-actin in human AD frontal cortex samples

Sample type			Sample size (*n*)	Correlation coefficient (*r*)	Significance (*P*)
AD	KIF5A	KIF5B	12	0.2657	0.4038
AD	KIF5A	KIF5C	12	0.8392	0.0006***
AD	KIF5B	KIF5C	12	0.5804	0.0479*
C/AD	KIF5A	KIF5B	12	0.6014	0.0386*
C/AD	KIF5A	KIF5C	12	0.8322	0.0008***
C/AD	KIF5B	KIF5C	12	0.5315	0.0754

To confirm our findings, we alternatively normalized the levels of KIF5 members to GAPDH, another often-used loading control. As with the β-actin control, the level of fl-hAPP did not distinguish AD from C/AD, nor did the levels of KIF5 members differ. Also, the levels of KIF5 members were significantly correlated with each other in the AD plus C/AD samples (Supplemental Figure 1A-D; Table 5); separately, the AD and the C/AD also demonstrated this correlation except for only a trend to significance between KIF5B and KIF5C in C/AD (Table 5). To more widely sample the AD brain, we processed the AD temporal cortex, another disease-involved region, and the cerebellum (Table 3). As for the frontal cortex, the levels of KIF5 members were not different from C/AD controls in either the temporal cortex or the cerebellum (Supplemental Figure 2A-E, G-K). In the temporal cortex, the levels of KIF5 members in the AD plus C/AD samples were significantly correlated with each other (Supplemental Table 1); however, in the C/AD, only KIF5A and KIF5C were correlated; in AD samples only KIF5B and KIF5C were correlated (Supplemental Table 1). In the cerebellum, positive correlations were detected in the combined AD and C/AD, AD alone, and C/AD alone. We note the possibility that a limited number of samples of these regions may have compromised the ability to demonstrate correlations in the separate AD and C/AD groups in the temporal cortex. Taken together, the findings are evidence against a change in KIF5 protein levels in AD. Moreover, co-regulation of KIF5 members in the frontal cortex is supported.

**Table 5:** Spearman’s correlation analysis between KIF5 members when normalized to GAPDH in human AD frontal cortex samples

Sample type			Sample size (*n*)	Correlation coefficient (*r*)	Significance (*P*)
C/AD and AD	KIF5A	KIF5B	24	0.6113	0.0015**
C/AD and AD	KIF5A	KIF5C	24	0.8991	< 0.0001***
C/AD and AD	KIF5B	KIF5C	24	0.6252	0.0011**
AD	KIF5A	KIF5B	12	0.6084	0.0358*
AD	KIF5A	KIF5C	12	0.8462	0.0005***
AD	KIF5B	KIF5C	12	0.7413	0.0058**
C/AD	KIF5A	KIF5B	12	0.6713	0.0168*
C/AD	KIF5A	KIF5C	12	0.9301	< 0.0001***
C/AD	KIF5B	KIF5C	12	0.5455	0.0666

### KIF5 levels were not changed in the AD-DS brain

**Fig. 3:**
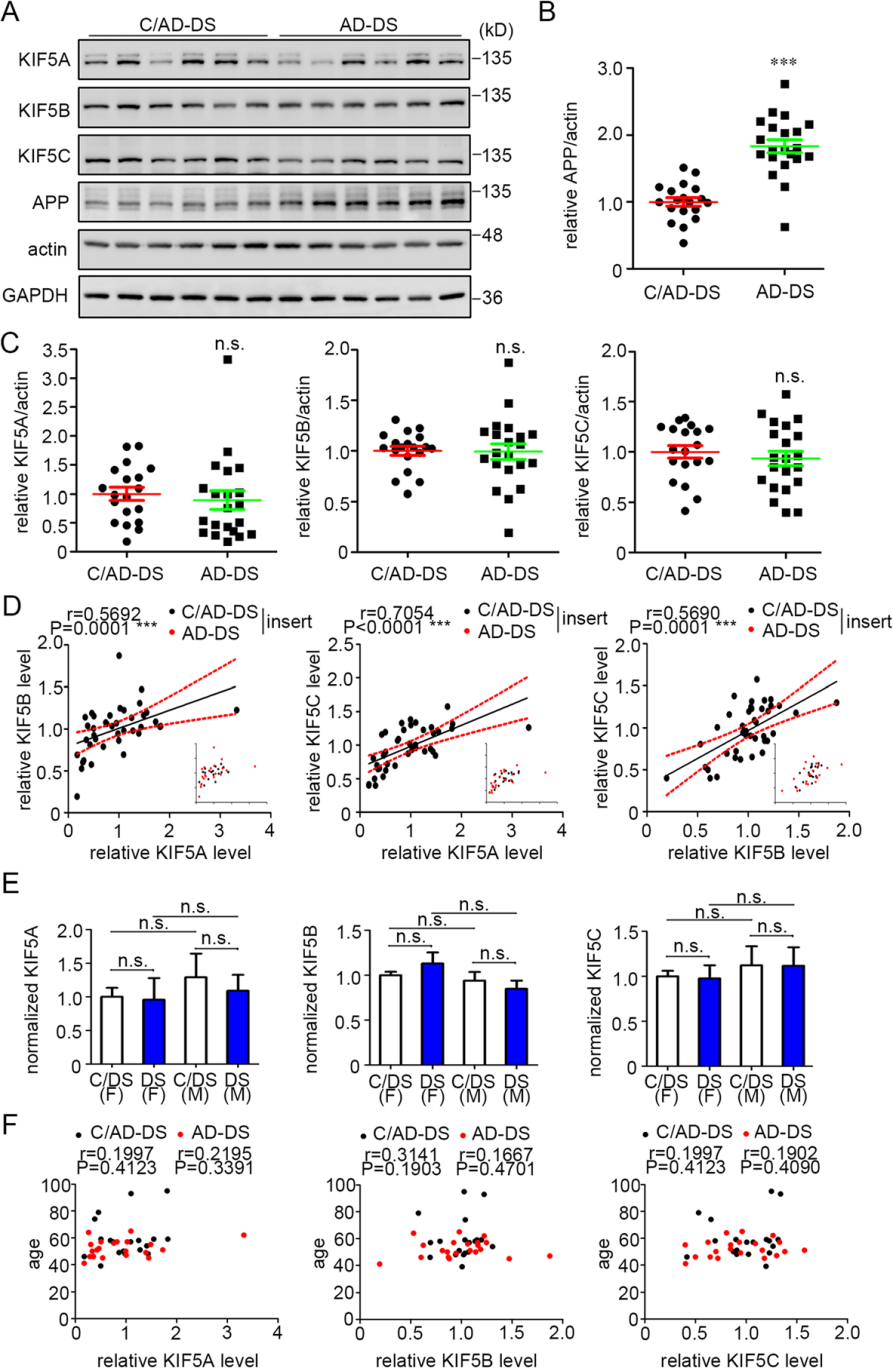
KIF5 levels were not changed in the frontal cortex of AD-DS brain. **a** Western blotting analysis of KIF5 levels in protein extracts from the frontal cortex of patients with AD-DS and C/AD-DS. fl-hAPP was also probed. β-Actin and GAPDH were each used as internal loading controls. **b** Quantitation and statistical analysis of the levels of fl-hAPP in AD-DS and C/AD-DS. **c** Quantitation and statistical analysis of the levels of KIF5 family members in AD-DS and C/AD-DS. **d** Spearman’s correlation analysis of KIF5 members in the combined C/AD-DS and AD-DS samples, with the red dotted line marking the 95% confidence interval. Insert with the same diagram indicates the distribution of C/AD-DS and AD-DS samples. **e** Quantitation and statistical analysis of the levels of KIF5 family members in females and males from AD-DS (DS) and C/AD-DS (C/DS). **f** Spearman’s correlation analysis between age and the levels of KIF5 members in AD-DS and C/AD-DS. *n* = 19 and 21 for C/AD-DS and AD-DS patients, respectively, Mann-Whitney test in **b**, **c**, and **e**. ***P < 0.001 unless stated. n.s., non-significant; F, female; M, male

The sharing of pathological hallmarks for AD and AD-DS prompted the evaluation of KIF5 members in the AD-DS brain (Fig. 3). As above, proteins were extracted from the frontal cortex of AD-DS and controls for AD-DS (C/AD-DS) and KIF5 family members, and fl-hAPP levels were evaluated by Western blotting (Table 2). Consistent with the fact that the majority of adults with DS show the increased expression of the *APP* gene with increases in the levels of fl-APP [18, 19, 21], and as previously reported [55, 56], we detected a significant increase in fl-hAPP normalized to β-actin in AD-DS brains relative to that in C/AD-DS (Fig. 3a, b). The average increase approximated that expected on the basis of increased gene dose. As for AD, the levels of KIF5 members normalized to β-actin in AD-DS subjects were not significantly different from the C/AD-DS controls (Fig. 3a, c). Also, in the combined AD-DS and C/AD-DS samples, the levels of KIF5 family members were strongly correlated with one another (Fig. 3d). The AD-DS samples alone demonstrated the positive correlation among KIF5 members; the C/AD-DS sample alone also showed the correlation except for KIF5A with KIF5B (Table 6). When we examined C/COMB, a sample containing all the controls, all KIF5 members were correlated and the significance of the correlations was increased, pointing to the benefit of increasing sample number (Table 6) and justifying the use of the C/COMB for the studies reported below. Normalizing to GAPDH, there was also a significant increase in fl-hAPP and no change in KIF5 family members in AD-DS, and the same results were obtained examining KIF5 correlations (Supplemental Figure 1E-H; Table 7). Finally, the levels of KIF5 family members were not a function of either gender (Fig. 3e) or age (Fig. 3f) for either AD-DS or C/AD-DS. In demonstrating normal KIF5 member levels and their correlated expression, our findings draw additional molecular parallels between AD and AD-DS.

**Table 6:** Spearman’s correlation analysis between KIF5 members when normalized to β-actin in human AD-DS frontal cortex samples

Sample type			Sample size (*n*)	Correlation coefficient (*r*)	Significance (*P*)
AD-DS	KIF5A	KIF5B	21	0.7143	0.0003***
AD-DS	KIF5A	KIF5C	21	0.6059	0.0036**
AD-DS	KIF5B	KIF5C	21	0.6057	0.0036**
C/AD-DS	KIF5A	KIF5B	19	0.3632	0.1265
C/AD-DS	KIF5A	KIF5C	19	0.6947	0.0010***
C/AD-DS	KIF5B	KIF5C	19	0.5684	0.0111*
C/COMB	KIF5A	KIF5B	21	0.5013	0.0206*
C/COMB	KIF5A	KIF5C	21	0.7688	< 0.0001***
C/COMB	KIF5B	KIF5C	21	0.6584	0.0012**

**Table 7:** Spearman’s correlation analysis between KIF5 members when normalized to GAPDH in human AD-DS frontal cortex samples

Sample type			Sample size (*n*)	Correlation coefficient (*r*)	Significance (*P*)
C/AD-DS and AD-DS	KIF5A	KIF5B	40	0.4850	0.0015**
C/AD-DS and AD-DS	KIF5A	KIF5C	40	0.6572	< 0.0001***
C/AD-DS and AD-DS	KIF5B	KIF5C	40	0.6225	< 0.0001***
AD-DS	KIF5A	KIF5B	21	0.6301	0.0029**
AD-DS	KIF5A	KIF5C	21	0.8150	< 0.0001***
AD-DS	KIF5B	KIF5C	21	0.6992	0.0006***
C/AD-DS	KIF5A	KIF5B	19	0.2965	0.2177
C/AD-DS	KIF5A	KIF5C	19	0.4825	0.0364*
C/AD-DS	KIF5B	KIF5C	19	0.4789	0.0380*
C/COMB	KIF5A	KIF5B	21	0.4805	0.0236*
C/COMB	KIF5A	KIF5C	21	0.5088	0.0156*
C/COMB	KIF5B	KIF5C	21	0.5178	0.0136*

We were intrigued by the positive correlations between the levels of KIF5 family members in the AD and AD-DS brains. To further explore this topic, we asked if the same correlations could be demonstrated at the level of the mRNAs for these proteins. For these studies, we elected to measure the mRNA levels of KIF5 family members in randomly selected samples (4 C/AD-DS and 5 AD-DS) (Supplemental Table 2). Individual samples demonstrated roughly comparable relative levels of the mRNAs for each of the KIF5 members (Supplemental Figure 3A), and there were significant positive correlations for family members with a trend to significance between KIF5B and KIF5C across the full sample set (Supplemental Figure 3B). These findings add additional support for possible co-regulation of KIF5 family members and suggest that regulatory events may act at the level of transcription and/or the stability of KIF5 mRNAs.

### Examining correlations between fl-hAPP and KIF5 in AD and AD-DS

An earlier report indicated that APP levels were inversely correlated with KIF5A protein levels in AD [48]. Given that KIF5 levels were not reduced in AD samples in our study, we elected to reevaluate this issue. Normalizing to β-actin when the AD and C/AD samples were taken together, we observed a positive correlation between fl-hAPP levels and two of the KIF5 members (KIF5A and KIF5B) with a trend in KIF5C (Supplemental Table 3A). Separately examining AD cases, we found that the correlation between fl-hAPP and KIF5C was unchanged but that those with KIF5A and KIF5B were absent. We then performed the same analysis after normalizing for GAPDH; combining the AD and C/AD samples showed significant correlations between fl-hAPP and each KIF5 member (Supplemental Table 3B). Separating the AD cases, the positive correlations persisted and indeed were stronger than when normalizing to β-actin (Supplemental Table 3B). When normalizing to β-actin, all KIF5 members were highly correlated with fl-hAPP in C/COMB; when normalized with GAPDH, the C/COMB showed a correlation for KIF5A and a trend for KIF5C (Supplemental Table 3A, B). Examining the AD-DS samples and normalizing to β-actin, the AD-DS and C/AD-DS samples taken together showed a trend to a positive correlation between fl-APP and KIF5B and KIF5C (Supplemental Table 4A). Independently examining AD-DS, fl-hAPP was significantly positively correlated with all KIF5 members when normalizing with β-actin (Supplemental Table 4A); fl-hAPP was also significantly correlated with KIF5 members in AD-DS when normalizing with GAPDH (Supplemental Table 4B).

Taken together, these data are evidence of correlations between fl-hAPP and KIF5 members in AD, AD-DS, and controls. These findings raise the possibility that mechanisms exist that links KIF5 and fl-APP. Additional studies will be needed to confirm and more fully explore this link.

### KIF5 family members were unchanged in the brains of young and aged Dp16 mice

**Fig. 4:**
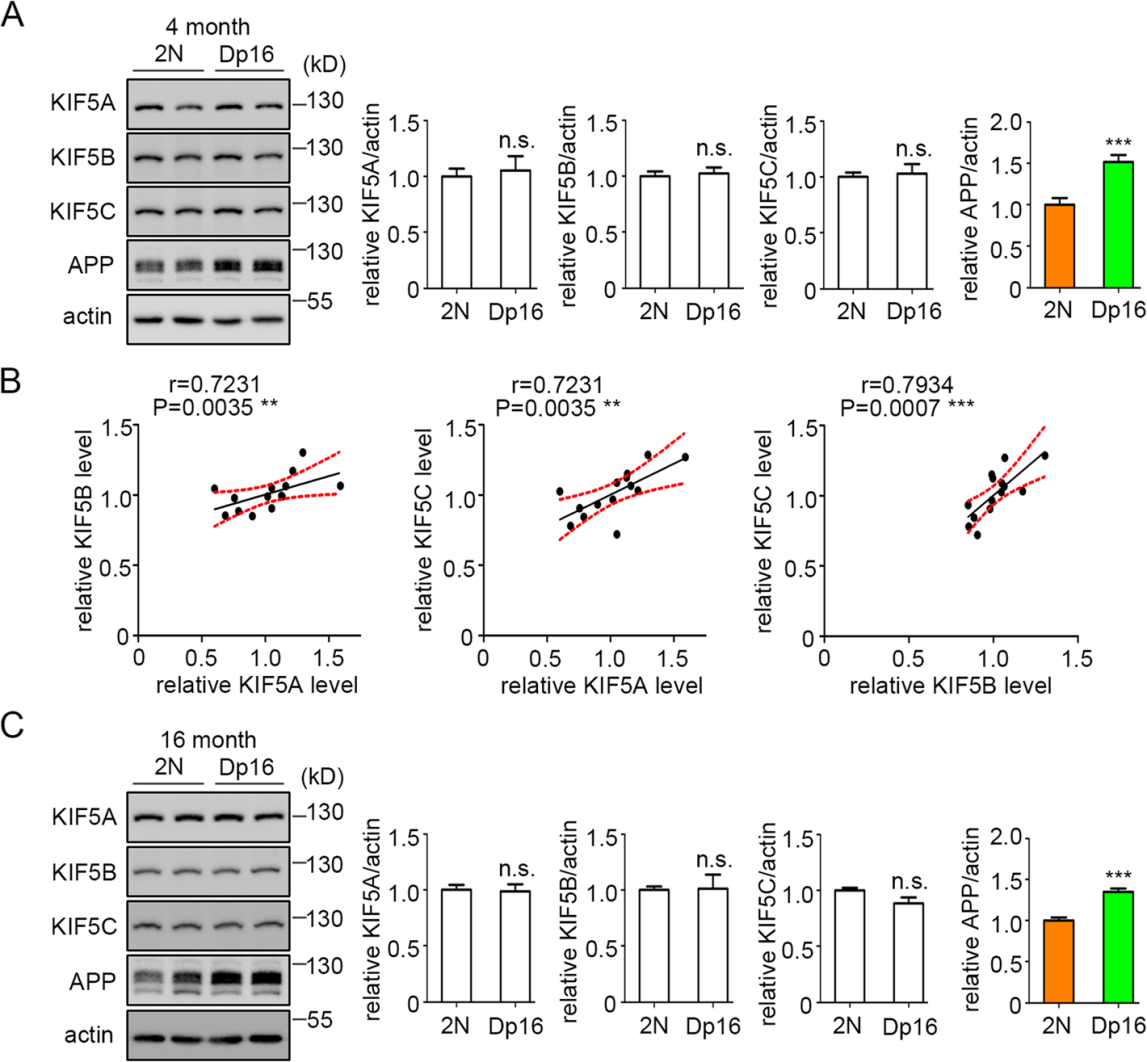
The levels of KIF5 family members did not differ from controls in the brains of young and aged Dp16 mice. Western blotting analysis of the levels of KIF5 family members in the brains from young (4 m, **a**) and aged (16 m, **c**) Dp16 mice. fl-mAPP was also probed; β-actin was used as a loading control. The left panels show representative immunoblots, while the right panels display the quantitation and statistical analysis. **b** Spearman’s correlation analysis of KIF5 members in the 2N and Dp16 samples taken together (4 months old). Mann-Whitney test. *n* = 4–9 mice for each category in **a** and **c** and *n* = 14 in **b** (7 2N and 7 Dp16); ***P < 0.001 unless stated. n.s., non-significant

The Dp16 mouse model of DS enables studies to explore the molecular and cellular mechanisms induced by the presence of an extra copy of many of the mouse genes whose human homologs are present on chromosome 21. To ask if this model replicated the finding that KIF5 family members are unchanged in AD-DS, we prepared brain lysates for Western blotting from Dp16 mice and its euploid (2N) controls; the forebrains only were examined, excluding the olfactory bulb and cerebellum. At age 4 months, as expected given the increase in mouse *App* gene dose, an increase in full-length mouse APP (fl-mAPP) was detected. Consistent with the study of AD-DS samples, we failed to find the differences for KIF5 family members (Fig. 4a). As with the human samples, in the combined 2N and Dp16 samples, KIF5 members were positively correlated with one another (Fig. 4b). Because, like the Ts65Dn mouse model of DS [37, 57], Dp16 mice display age-related neuropathology ([50, 58] and WCM, unpublished data), we asked if there would be age-related changes in KIF5 levels by examining the levels of KIF5 family members in aged Dp16 mice (16 months). As with younger mice, fl-mAPP levels were increased in Dp16 mice; again, there was no difference for KIF5A, KIF5B, and KIF5C in comparison with age-matched 2N mice (Fig. 4c). Finally, we asked if the correlations between KIF5 members and fl-APP were replicated in the mouse. We discovered no correlations between these measures in either 2N and Dp16 (Supplemental Table 5). We conclude that while mice share with humans with DS the absence of changes in KIF5 levels and possible coordinate regulation of KIF5 members, they fail to show a correlation between fl-mAPP and KIF5 levels.

### KIF5 levels are not changed by either APP OE or KO in mouse brains

**Fig. 5:**
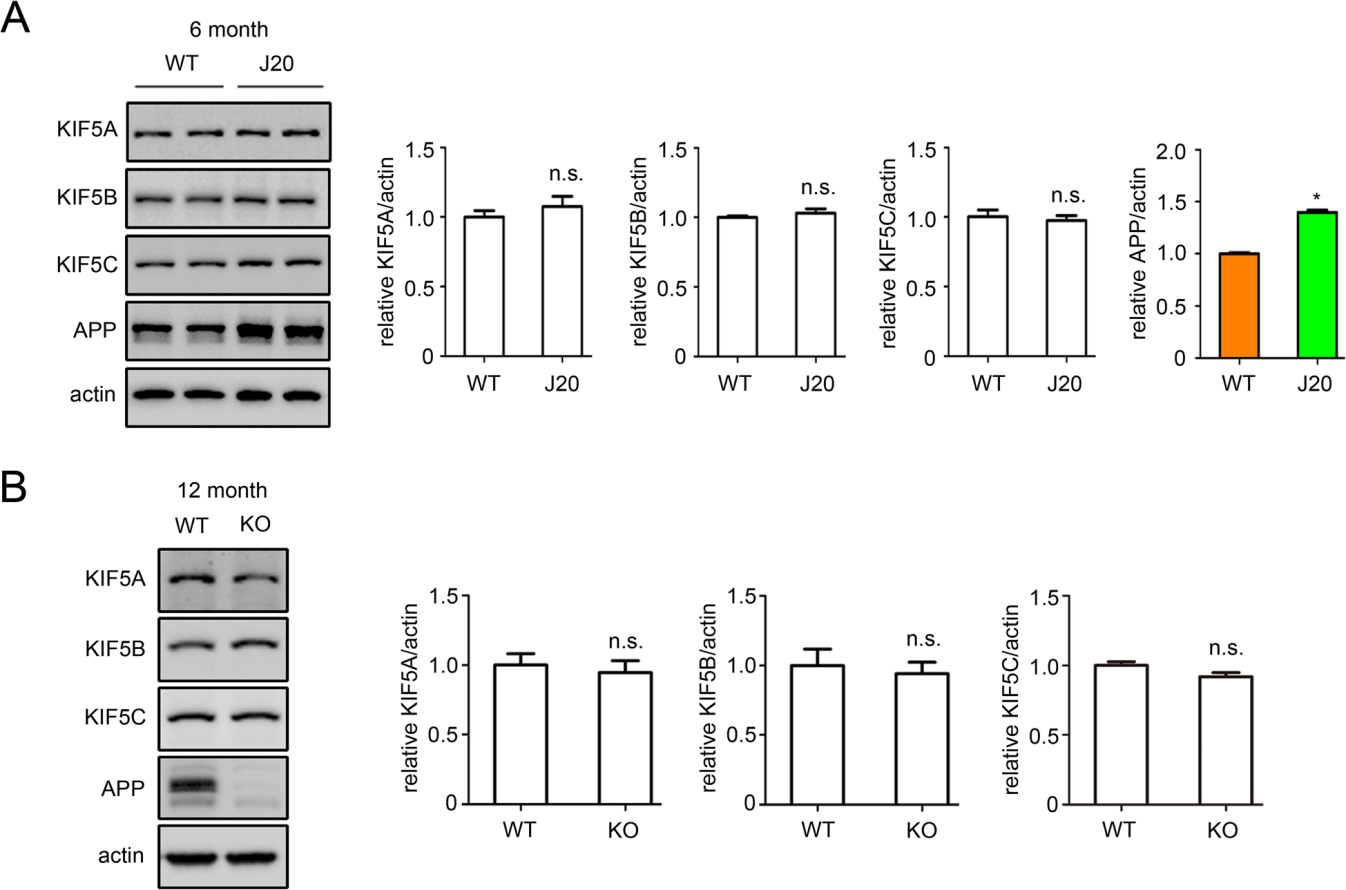
The levels of KIF5 family members were not changed in the J20 mouse or the APP KO mouse brain. **a** As in Fig. 5, Western blotting was used to measure the levels of KIF5 family members in the brains of 6-month-old J20 mice and their age-matched controls. APP was also probed; β-actin was used as a loading control. **b** The levels of KIF5 members were examined in the brains of 12-month-old *APP* KO mice and their age-matched control wild-type mice. The left panels show representative immunoblots, while the right panels display the quantitation and statistical analysis. Mann-Whitney test. *n* = 4–5 for each; *P < 0.05. n.s., non-significant

In view of an interest in defining a mouse model system that allows one to either refute or confirm the lack of changes in KIF5 in the setting of Aβ-linked amyloid pathology, we analyzed KIF5 in the brains of J20 mice. The latter is a well-established AD model in which overexpression (OE) of human APP carrying the Swedish and Indiana mutations [59] results in amyloid plaques and other AD-relevant pathology by age 6 months [59–62]. We extracted total protein from the whole brains of age-matched controls and J20 mice at 6 months. Relevant to an earlier report focused on Aβ effects on anterograde transport [43], J20 mice produce relatively more Aβ42 than mice expressing comparable amounts of wild-type human APP [59]. As in our human samples, we found no difference between J20 and age-matched wild-type mice in any KIF5 member (Fig. 5a). Finally, to exclude any impact of *APP* gene expression on KIF5 levels, we examined KIF5 levels in the brains of *APP* knockout (KO) mice at age 12 months. Again, no alteration of KIF5 members was detected, as compared with the age-matched wild-type mouse (Fig. 5b). We conclude that KIF5 levels are not impacted in a mouse brain in which Aβ levels are increased, even if amyloid plaques are present. These findings point to the conservation of control levels of KIF5 in humans with AD and AD-DS and in corresponding mouse models.

### KLC1 was reduced in both AD and AD-DS brains but not in the Dp16 and J20 mice

**Fig. 6:**
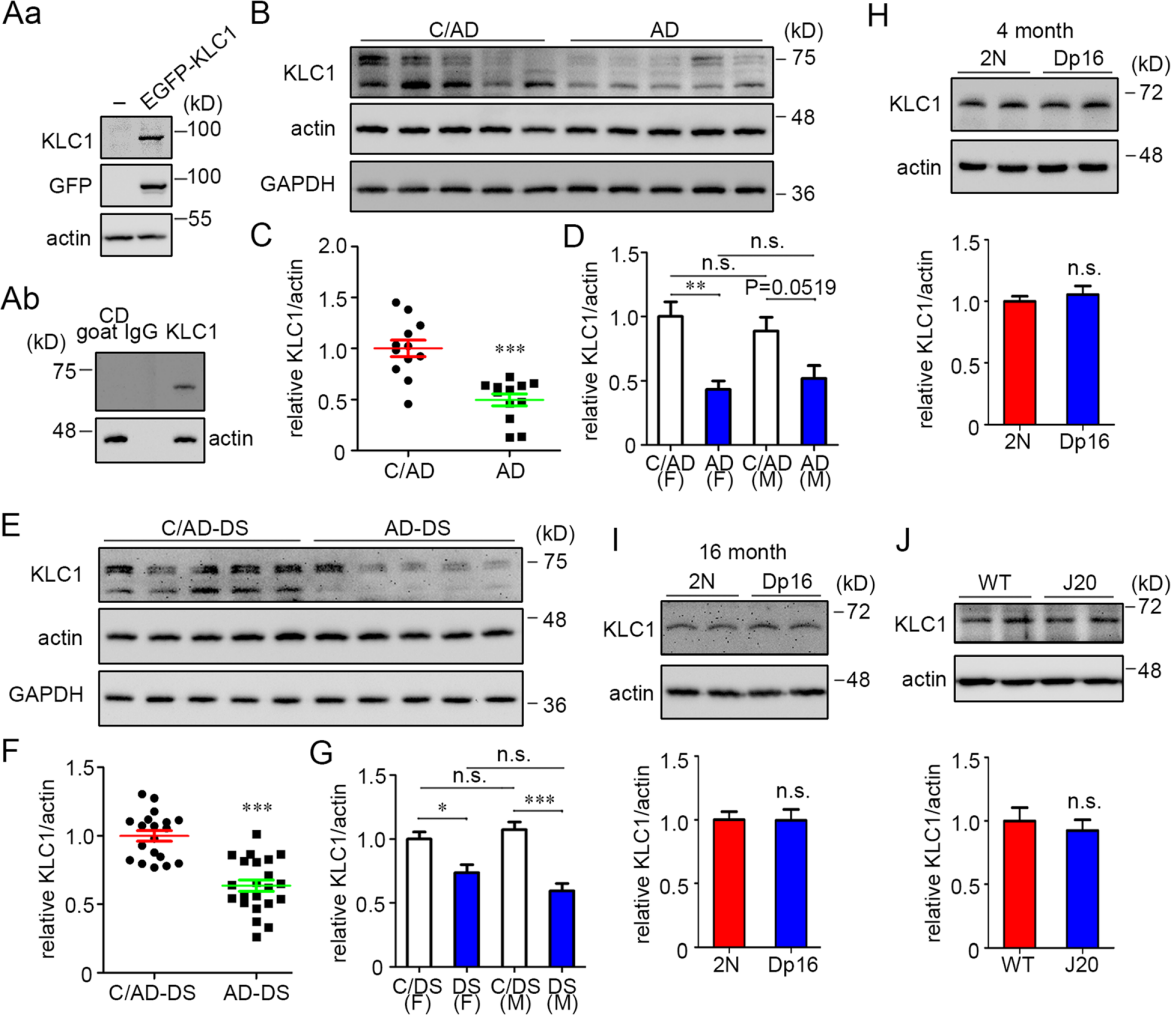
KLC1 was reduced in the frontal cortex of both AD and AD-DS brains, but not in the brains of Dp16 or J20 mouse models. **Aa** EGFP-KLC1 was transiently expressed in HEK293T cells for 36 h; after cell lysis, the soluble proteins were submitted to Western blotting to evaluate the specificity of the KLC1 antibody. **Ab** A goat IgG antibody to cathepsin D (CD) was used to further test the specificity of the KLC1 antibody in mouse brain lysate. **B** Western blotting analysis of KLC1 levels in protein extracts from the frontal cortex of patients with AD and from C/AD. For quantitation, the intensity of the doublet band at 75 kD and the band at ~ 65 kD were combined. **C** Quantitation and statistical analysis of KLC1 in AD and C/AD. **D** Statistical analysis of KLC1 levels in females and in males from AD and C/AD. **E** Western blotting of KLC1 levels in protein extracts from the frontal cortex of patients with AD-DS and C/AD-DS. **F** Quantitation and statistical analysis of the levels of KLC1 in AD-DS and C/AD-DS. **G** Statistical analysis of the levels of KLC1 in both females and males from AD-DS and C/AD-DS. **H**–**J** Western blotting analysis of KLC1 levels in the brains from 4- or 16-month-old Dp16 and 6-month-old J20 mice. *n* = 3–7 mice for each category. β-Actin or GAPDH was employed as an internal control for all panels. Mann-Whitney test; *P < 0.05, **P < 0.01, ***P < 0.001. n.s., non-significant; F, female; M, male

KLC1 is essential for anterograde transport of many cargoes and was reported to be downregulated in some, but not all, studies of AD [45–48]. To further explore possible shared molecular changes in AD and AD-DS, we measured KLC1 levels. The KLC1 is enriched in neuronal tissue [33]. KLC has several isoforms with molecular weight varying from 51 to 76 kD [63]. First, we confirmed the specificity of the KLC1 antibody by probing blots of the lysates of HEK293T cells transfected with EGFP-KLC1. A band corresponding to the molecular weight of the fusion protein was only present in the lysates of transfected cells (Fig. 6Aa). A major band detected by the KLC1 antibody was found at approximately the calculated molecular weight (65–70 kD) in mouse brain lysate. No signal was detected using a goat IgG antibody to an unrelated antigen (cathepsin D)(Fig. 6Ab).

In human brain samples, bands at apparent molecular weights of between 63 and 75 kD were detected and were quantitated together. In AD samples, as compared with age-matched controls, we found a significant ~ 50% reduction in KLC1 levels when normalized to β-actin (Fig. 6B, C). Reduced KLC1 was seen in both AD females and males (Fig. 6D). The change in the levels of KLC1 in the frontal cortex of AD was confirmed when the levels of KLC1 were normalized to GAPDH (Fig. 6B; Supplemental Figure 1I). Interestingly, in the AD temporal cortex and cerebellum tissues, we failed to find a reduction in KLC1, as compared with C/AD (Supplemental Figure 2A, F, G and L), pointing to a region-specific change in the levels of KLC1. The finding in the AD cerebellum is consistent with a prior report [47]. KLC1 levels were also decreased in AD-DS, with an average reduction of ~ 40% when normalized to either β-actin or GAPDH (Fig. 6E, F; Supplemental Figure 1J). There was no significant effect of gender on the levels of KLC in C/AD-DS or in AD-DS samples (Fig. 6G). Importantly, we found a significant negative correlation between PMI and KLC1 in AD and AD-DS brains, but not in control brains (Supplemental Table 6). We then analyzed a subset of samples with a very short PMI (≤ 6 h); under this condition, PMI was not significantly correlated with the levels of KLC1 in either AD (*r* = − 0.2060; *P* = 0.7139) or AD-DS (*r* = − 0.5786; *P* = 0.0622) samples. In these very short PMI samples, KLC1 was again significantly reduced in both AD and AD-DS brains compared with control brains (Supplemental Figure 4). Pointing again to the differences between human samples and those in mouse models, we failed to find a difference in the levels of KLC1; KLC1 levels were equivalent to control values in both the Dp16 and J20 brains (Fig. 6H–J). We conclude that KLC1 levels are reduced in both males and females with AD and AD-DS.

The reduction of KLC1 protein in AD-DS raised the question as to whether this change was solely detected at the protein level or was also registered at the level of the mRNA for KLC1. A prior report found that KLC1 mRNA levels were unchanged [47]. To address this question, we quantitated the levels of KLC1 mRNA in the same randomly chosen set of AD-DS and C/AD-DS cases used for comparing the levels of KIF5 mRNAs. No change in KLC1 mRNA was found (Supplemental Figure 5). This finding suggests that differences in translation or degradation of KLC1 underlie the reductions seen in AD and AD-DS.

### Examining correlations between KLC1, KIF5 members and fl-APP

Next, we examined possible relationships between the levels of KIF5 levels and KLC1, a topic addressed in the earlier publication that pointed to a positive correlation [48]. Normalizing to β-actin, we failed to find correlations for KIF5 members and KLC1 in the AD and AD-DS samples; a single positive correlation in the C/COMB samples was absent when normalizing with GAPDH (Supplemental Table 7A, B). When we tested for correlations between KLC1 and fl-hAPP and normalized with β-actin, we found significant positive correlations for C/COMB and for AD, but not for AD-DS (Supplemental Table 7A); when normalizing with GAPDH, only the correlation in C/COMB remained (Supplemental Table 7B). Finally, as for the human samples, while KLC1 was not correlated with KIF5 family members in either 2N or Dp16 brains, it was correlated with fl-mAPP in 2N brains (Supplemental Table 7A). These findings are evidence against consistent correlations between KLC1 and KIF5 members or between KLC1 and fl-hAPP in the AD and AD-DS brains.

## Discussion

Disruption of axonal transport has been suggested as contributing to the pathogenesis of neurodegenerative disorders including AD and DS [1, 17, 64, 65]. The essential roles played by axonal transport point to this facet of neuron function as a potential source of vulnerability to degeneration. Many factors can be envisioned to contribute to deficits in axonal transport: abnormal microtubule dynamics/stability, deficits in essential cargoes, deficiencies in adaptor proteins that scaffold cargoes for loading onto motor proteins, and abnormalities in the levels/functions of motor proteins and complexes responsible for transporting cargoes to and from cell bodies.

It is interesting that KIF5 family members have been shown to be connected to many neurodegenerative disorders. KIF5A along with other kinesin members including KIF1B and KIF21B were significantly reduced at the mRNA level and KIF5A at the protein level in gray matter of multiple sclerosis (MS) brains [66]. In another study, KIF5A was reduced in MS white matter, and individuals carrying certain MS risk alleles demonstrated decreased levels of KIF5A [67]. Changes in KIF21B have also been reported in MS and AD [68]. In addition to changes in expression of KIF5 proteins, mutations in the KIF5A gene were associated with several neurodegenerative diseases including hereditary spastic paraplegias (SPG10), Charcot-Marie-Tooth type 2 (CMT2), amyotrophic lateral sclerosis-frontotemporal dementia (ALS-FTD), and MS, supporting a significant role for KIF5 family members, including KIF5A, in disorders implicating disrupted axonal transport [69–73].

There is little data for which, if any, proteins that support axonal transport contribute to neuron dysfunction and loss in AD and AD-DS, but recent studies pointed to changes in the levels of motor proteins or adaptor proteins. Thus, changes in both KIF5A and KLC1 in AD have been reported [43, 45–47]. Though changes in either could disrupt anterograde axonal transport, these earlier reports were inconsistent and conflicting [42, 43, 47, 48].

In this study, we examined the levels of KIF5 family members and KLC1 in both AD and AD-DS brains and normalized to both β-actin and to GAPDH. No changes were detected in the average level of KIF5 family members in AD and AD-DS in comparison with controls, nor were there changes in KIF5 members comparing males to females or across the age span in AD or AD-DS. Interestingly, evidence for coordinate regulation of KIF5 family members was discovered in AD, AD-DS, and control sample sets. Positive correlations between KIF5 members and the level of fl-APP were also detected for AD-DS, AD, and control samples. Importantly, in contrast to the findings for KIF5, both the AD and AD-DS frontal cortex samples showed significant reductions in KLC1 in both males and females, including a subset of samples with a PMI shorter than that shown to be correlated with degradation of KLC1. These findings raise the possibility of reductions in the anterograde transport of selected cargoes in AD and AD-DS and prompt additional studies to test this hypothesis.

Our findings for KIF5 family members in AD are at odds with recently published work [43]. Several possibilities exist to explain the difference in findings. First, while brain region-specific changes of KIF5A may contribute, our studies sampling both frontal and temporal cortex showed no reductions. Thus, our findings are at odds with a previous study that examined KIF5 levels in the temporal cortex [43]. Sample quality is another factor to be considered. We noted significant variability across samples in the levels of KIF5B and KIF5C, especially for KIF5A. As a speculation, differences in the stability of KIF5 proteins in postmortem samples and longer PMIs could contribute to the differences across studies [74]. We employed samples with a far shorter average PMI for both AD and controls than for earlier studies demonstrating either a decrease in KIF5A [43] or an increase in KIF5A [42] (Supplemental Table 8). Importantly, we were able to show that PMIs as long as 23 h had no obvious effects on the levels of any KIF5 members (Supplemental Table 6). Samples with PMIs as long as 72 h were examined in earlier reports, possibly contributing to different findings [42, 48]. In contrast to the relative stability of KIF5 members, we found a significant negative correlation between PMI and KLC1 in AD and AD-DS brains but, interestingly, not in control brains. However, we confirmed the significant reduction of KLC1 in both AD and AD-DS samples with PMIs ≤6 h—i.e., under conditions in which there was no significant postmortem degradation of KLC1 in either AD or AD-DS samples. It is interesting, then, that the stability of KLC1 serves to distinguish control and disease brains. Another important consideration is the method for detecting changes. The use of dot blot technology is inherently less accurate for evaluating protein levels than Western blotting; this source of error is reduced when only a band of the expected molecular weight is quantitated. Finally, sample number is a consideration. While two earlier studies employed a large number of AD samples [42, 48], far fewer were examined in a study reporting a reduction in KIF5A and KIF5B (*n* = 4 for AD) [43] (Supplemental Table 8). We examined 12 AD, 21 AD-DS, and a total of 21 control samples. We conclude that differences in PMI and in analytical methods, and in one case the number of samples used, best account for the differences in findings between earlier studies and ours.

Using two different loading controls, β-actin or GAPDH, we observed correlations in the levels of KIF5 family members at the protein level in AD, AD-DS, and controls, evidence suggesting co-regulation among the three KIF5 family members. To exclude possible spurious correlations between ratios with a common divisor [75], we then re-examined the positive correlations by determining whether they persisted when one KIF5 member was normalized to β-actin and the other to GAPDH. Most positive correlations remained (Supplemental Table 9). To supplement these findings, we measured the mRNA levels of KIF5 members in AD-DS and C/AD-DS brains, finding that the levels of individual KIF5 members were highly correlated with one another. The mechanism underlying is yet to be defined but suggests regulation at the levels of mRNA. Interestingly, manipulation of any KIF5 member through gene knockout or siRNA-mediated knockdown had no obvious effects on the others [32, 76, 77]. These earlier findings are consistent with our findings that overexpression of each KIF5 member in transfected cell systems did not change the levels of the other members. Further studies will be needed to understand what are apparently different regulatory modes for endogenous and extrinsically induced changes in expression.

Given the increase in *APP* gene dose and APP and its products in DS, and earlier reports linking APP levels to KIF5A in AD, it was of interest to ask if APP protein levels were correlated with KIF5. Others found that fl-APP was negatively correlated with KIF5A levels in AD; however, the significance of this finding was uncertain, especially given that KIF5A levels were not a function of the Braak stage in AD [48]. Nor did the authors find a significant correlation between KIF5A and either soluble or insoluble Aβ species [48], even though another study argued for a role for Aβ in reducing KIF5A in AD [43]. Strikingly, we found a positive correlation between fl-APP levels and each KIF5 member in AD, AD-DS, and control samples, albeit with differences on the basis of which protein was used to normalize the samples. To test for possible spurious correlations, we re-examined samples showing positive correlations by determining whether they persisted when a KIF5 member was normalized to GAPDH and fl-APP was normalized to β-actin. Most positive correlations remained (Supplemental Table 9). Whether or not KIF5 levels and fl-APP levels are mechanistically linked is uncertain. APP itself is transported by the KIF5/KLC complex and can be coimmunoprecipitated by antibodies against all the KIF5 family members and KLC1 or KLC2 from mouse brain lysates [78]. Interestingly, compared with KIF5A, the association of KIF5B or KIF5C with APP depends to a greater extent on KLC1 than KLC2, reflecting the uniqueness of each KIF5 [78]. As a speculation, it is intriguing to suggest that fl-APP levels positively regulate the levels of KIF5 members. The discrepancies between our findings and others for KIF5 members and fl-APP are subject to some of the same considerations as for KIF5 members alone, as cited above.

KIF5 complexes with KLC subunit to engage in axonal transport [4]. The significance of this interaction is indicated by genetic deletion of KLC which results in early selective axonal transport deficits accompanied by axonopathies with cytoskeletal disorganization and abnormal cargo accumulation [79]. Axonal transport of APP was shown to be mediated by direct binding to KLC1 and APP axonal transport was significantly reduced in a KLC1 mutant mouse [78], suggesting an essential role for KLC1 in anterograde axonal transport of APP. Regulated movement of APP-containing vesicles has been described. For example, the JNK-interacting protein 1 (JIP1) has been shown to regulate APP axonal transport through binding to KLC1 [80]. Calsyntenin-1, a KLC ligand, has been linked to APP axonal transport and APP processing [81, 82]. Given these findings, the reported downregulation of KLC1 in AD brains is of considerable interest [45–47]. In our study, there was a significant marked reduction of KLC1 in the frontal cortex of both AD and AD-DS but not the temporal cortex and cerebellum. These data point to another shared element of pathogenesis for AD and AD-DS and to the possibility that reductions in KLC1 are responsible for disrupting the transport of KLC1-mediated movement of cargoes in these regions.

Comparing the findings in human samples with those in the brains of the Dp16 mouse model of DS and the J20 model of AD pointed to both similarities and differences. KIF5 levels were maintained at control levels in both models, and in the Dp16 model, the levels KIF5 family members were positively correlated. However, in contrast to human cases, there was no correlation between fl-APP levels and KIF5 levels in the Dp16 model, pointing to a potentially important difference in the regulation of KIF5 levels in the mouse. A more striking disconnect with human samples was that KLC1 levels were not reduced in either the Dp16 or J20 mouse. Whether subregional analysis would detect the differences in these models will require further study. At present, our findings suggest that models to explore KLC1-mediated changes in anterograde transport in the Dp16 or J20 mouse will require interventions to downregulate KLC1.

### Limitations

The use of postmortem human tissues poses limitations to findings and their interpretation. While there are clear benefits to such use, including the ability to underpin and thereby ground findings to allow for comparisons between model systems and humans, the quality of human postmortem samples is a function of the PMI. Extended PMIs result in degradation of molecules and cells; in the extreme case, the degree of degradation would invalidate findings and conclusions [74]. To address this limitation, we employed samples with a relatively short average PMI. Indeed, on average, it was well within the range reported to preserve the integrity of RNA [74], a species known to be especially susceptible to postmortem degradation. Moreover, we found no evidence of PMI-linked reductions in KIF5 members in control, AD, or AD-DS samples. Also, we note that PMI is not a factor for the mouse samples, which can be frozen rapidly, and that the levels of KIF5 family members in mouse brain were also positively correlated with one another, as was seen in human samples. Finally, we observed a significant negative correlation between KLC1 and PMI in AD and AD-DS, but not in control brains, and confirmed the reduction of KLC1 in AD and AD-DS samples with a very short PMI—i.e., using PMIs wherein there were no significant correlations in the levels of KLC1 in either AD or AD-DS.

A second limitation is the extent to which our findings speak to KIF5 and KLC1 levels in all AD-vulnerable human brain regions. We examined the levels in the frontal cortex of human AD and AD-DS samples and the temporal cortex and cerebellum of AD samples. We note that KIF5 family members were preserved in all regions and that KLC1 was reduced only in the frontal cortex. A more complete list of regions bearing AD pathology will be needed to further explore the existence of regional changes and their possible links to changes in axonal transport.

A third limitation is that while the reduction in KLC1 suggests there may be defects in anterograde transport, direct evidence demonstrating this will be required to prove the point. An important question is what model system would be appropriate for such a study. Because KLC1 was not changed in the mouse models of either AD or AD-DS, studies in mouse neurons in which KLC1 was knocked down would be needed to recapitulate this change. But it is possible that the cellular environment so created would differ considerably from that in human neurons. Studies in the Dp16 mouse could at least explore the reduction in KLC1 in a genetic environment in which there is increased gene dose for many of the mouse homologs of human genes on chromosome 21. Moreover, the Dp16 and related models may prove useful in exploring other factors that could impact axonal transport, including those that act through post-translational modification of KIF5s and KLC1 [45, 83]. Of possibly greater value would be the use of human in vitro models of induced pluripotent stem cell (iPSC)-derived control, AD and AD-DS neurons to determine whether or not KLC1 is reduced and, if so, the potential consequences for the transport of known cargoes, such as APP. Indeed, human neurons that model sporadic AD and AD-DS, as well as familial AD (FAD), may provide unique opportunities to determine whether or not axonal transport is compromised.

## Conclusion

In summary, we show that while KIF5 family members were not changed in AD or AD-DS, significant reductions were detected in KLC1 in a subset of samples with PMI (≤ 6 h) thus raising the possibility that disruption of anterograde axonal transport may be discovered for both conditions. Our results thus unveil novel shared molecular features in AD and AD-DS and point to the importance of further studies to explore the significance of changes in the Kinesin-1 complex in the pathogenesis of AD and AD-DS.

## Supplementary Information

**Additional file 1:.** Supplementary figures and tables.

## Data Availability

All data generated or analyzed during this study are included in this article.

## Abbreviations

RNAs: Ribonucleic acids
AD: Alzheimer’s disease
DS: Down syndrome
AD-DS: AD in DS
KIF5: Kinesin family member 5
KLC: Kinesin light chain
APP: Amyloid precursor protein
PD: Parkinson disease
HD: Huntington disease
ALS: Amyotrophic lateral sclerosis
NFTs: Neurofibrillary tangles
HSA21: Human chromosome 21
KHC: Kinesin heavy chain
mRNA: Messenger ribonucleic acid
fl-APP: Full-length APP
CTF: C-terminal fragment
PMI: Postmortem interval
GAPDH: Glyceraldehyde-3-phosphate dehydrogenase
fl-hAPP: Full-length human APP
fl-mAPP: Full-length mouse APP
OE: Overexpression
KO: Knockout
MS: Multiple sclerosis
CMT2: Charcot-Marie-Tooth type 2
ALS-FTD: Amyotrophic lateral sclerosis-frontotemporal dementia
JIP1: JNK-interacting protein 1
FAD: Familial AD
iPSC: Induced pluripotent stem cell
