# Supplementary material for: Normal levels of KIF5 but reduced KLC1 levels in both Alzheimer disease and Alzheimer disease in Down syndrome: evidence suggesting defects in anterograde transport
Source: Alzheimers Res Ther. 2021 Mar 10;13:59. doi: 10.1186/s13195-021-00796-6 (PMC7945332; doi:10.1186/s13195-021-00796-6)
Supplement: Supplementary file 1 — Additional file 1:. Supplementary figures and tables. [file 13195_2021_796_MOESM1_ESM.docx]

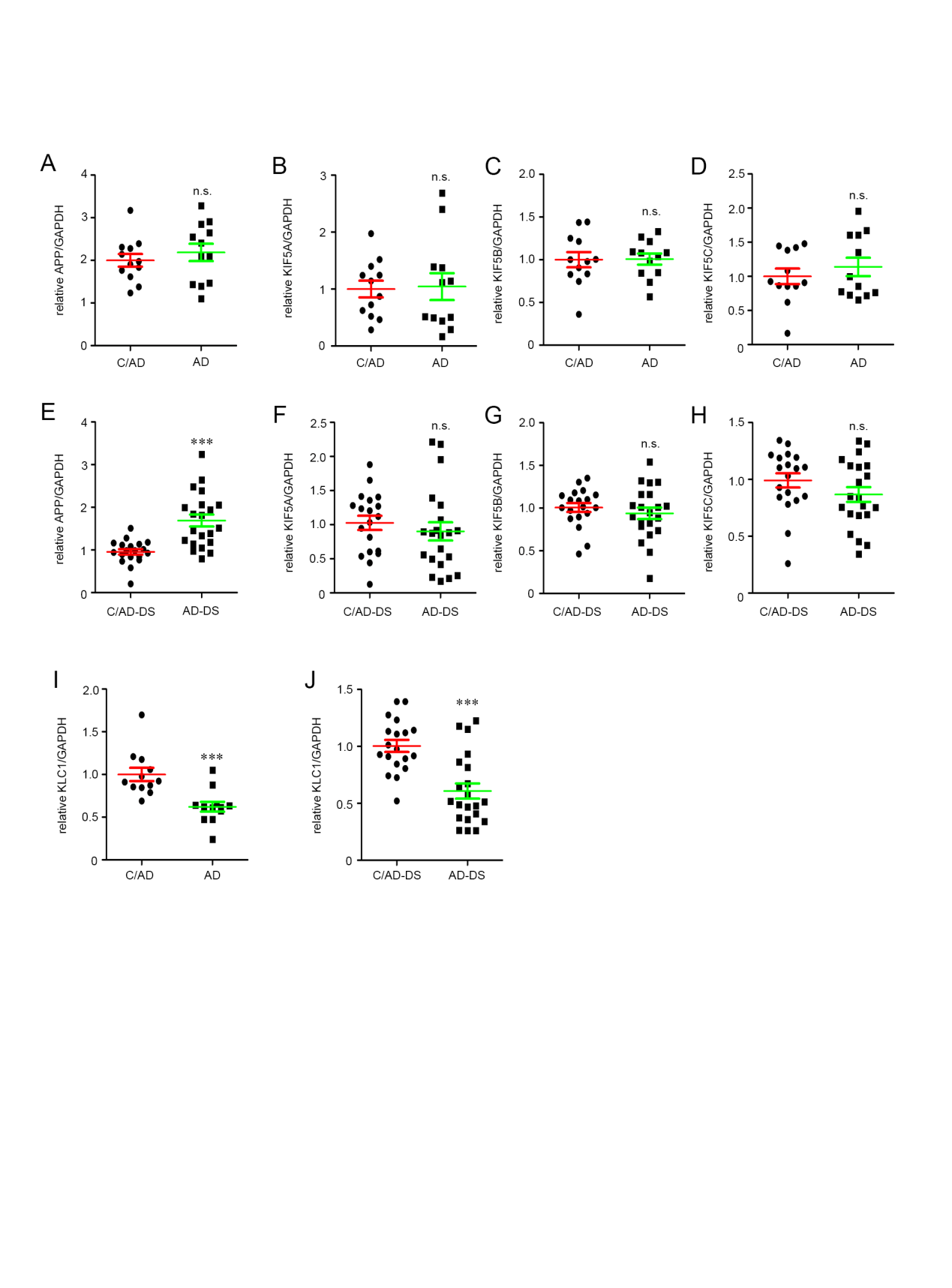


**Supplemental Fig. 1. The levels of fl-hAPP, KIF5 family members and KLC1 normalized to GAPDH in the frontal cortex of AD and AD-DS.** (A-D) Quantitation and statistical analysis of the levels of fl-hAPP and KIF5 family members in AD and C/AD. (E-H) Quantitation and statistical analysis of the levels of fl-hAPP and KIF5 family member in AD-DS and C/AD-DS. (I-J) Quantitation and statistical analysis of the levels of KLC1 in AD, AD-DS and controls. n=12 for both C/AD and AD patients; n=19 and 21 for C/AD-DS and AD-DS patients, respectively. Mann-Whitney test. ***P < 0.001; n.s. non-significant.


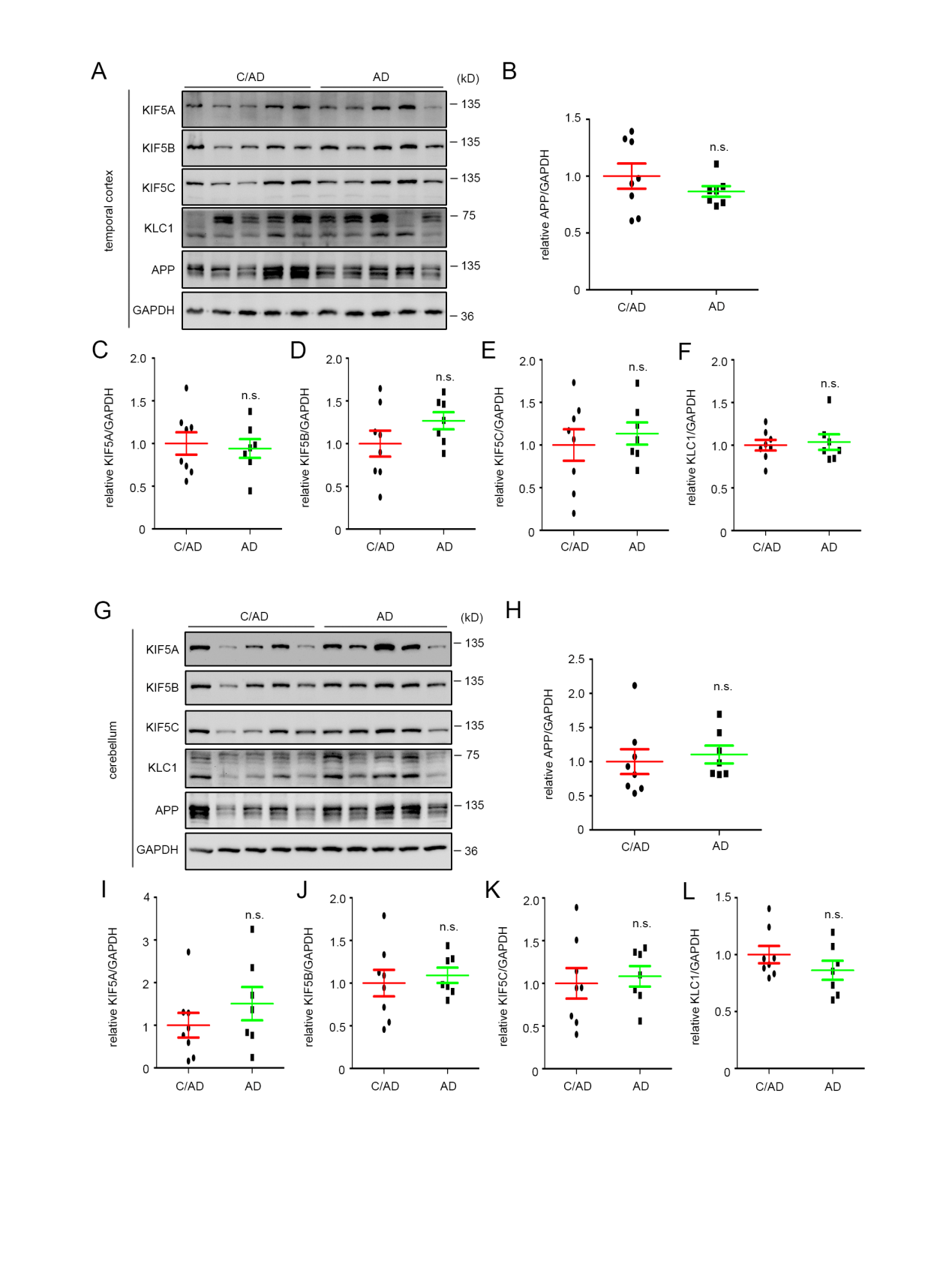


**Supplemental Fig. 2. The levels of fl-hAPP, KIF5 family members and KLC1 normalized to GAPDH in the temporal cortex and cerebellum of AD.** (A) Western blotting of KIF5 and KLC1 levels in protein extracts from the temporal cortex of patients with AD and C/AD. fl-hAPP was also probed. GAPDH served as loading control. (B-F) Quantitation and statistical analysis of the levels of fl-hAPP, KIF5 family members and KLC1 in the temporal cortex of AD and C/AD. (G) Western blotting of KIF5 and KLC1 levels in protein extracts from the cerebellum of patients with AD and C/AD. fl-hAPP was also probed. GAPDH was used as a loading control. (H-L) Quantitation and statistical analysis of the levels of fl-hAPP, KIF5 family members and KLC1 in the cerebellum of AD and C/AD. n=8 for C/AD, n=7 for AD patients. n.s. non-significant.


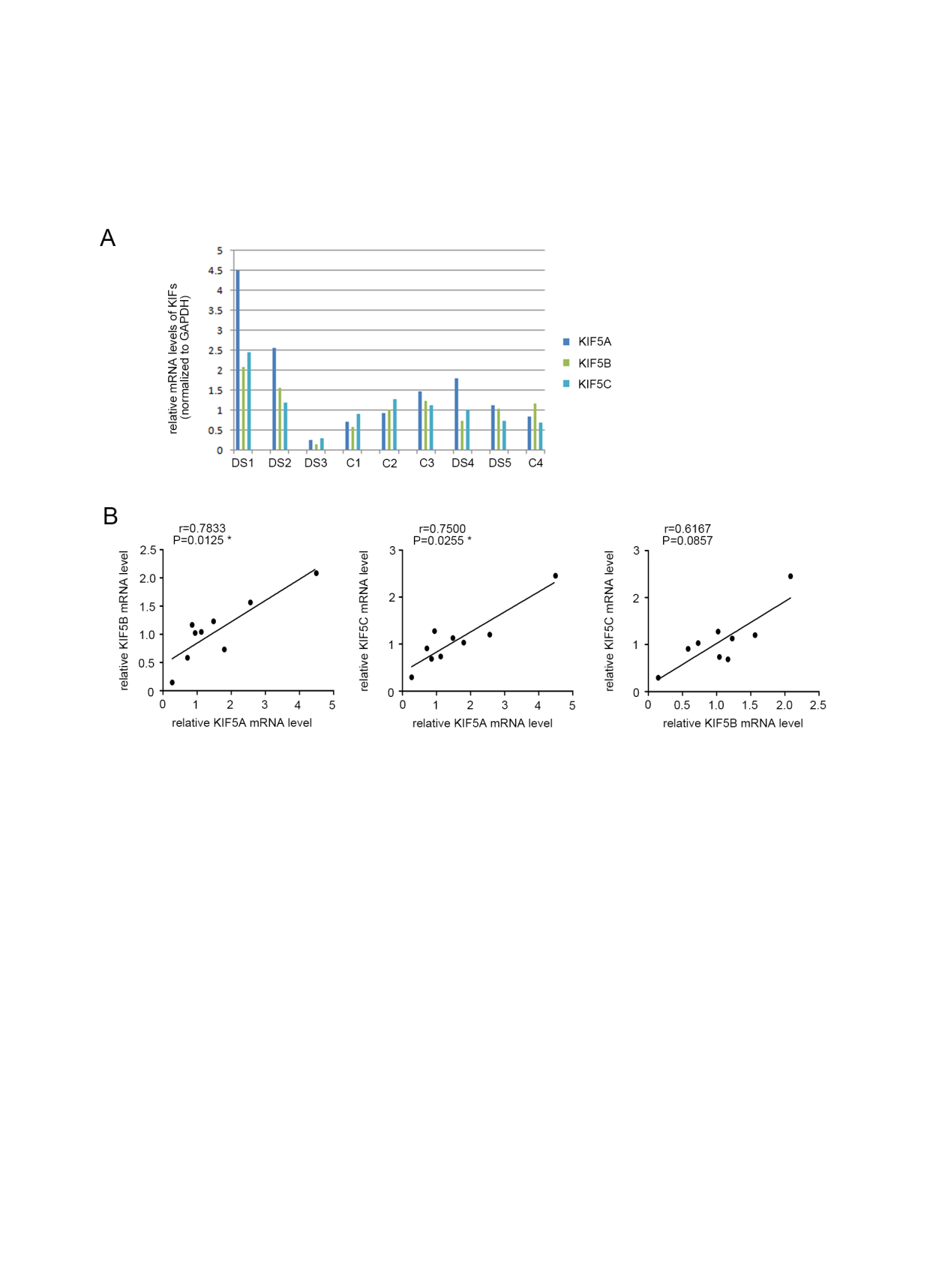


**Supplemental Fig. 3. The mRNA levels for KIF5 family members in the frontal cortex of AD-DS were well correlated.** (A) The relative mRNA levels of KIF5 family members from 9 randomly selected samples [4 C/AD-DS (C) and 5 AD-DS (DS)]. (B) Spearman’s correlation analysis of KIF5 members in combined C/AD-DS and AD-DS samples as in A.


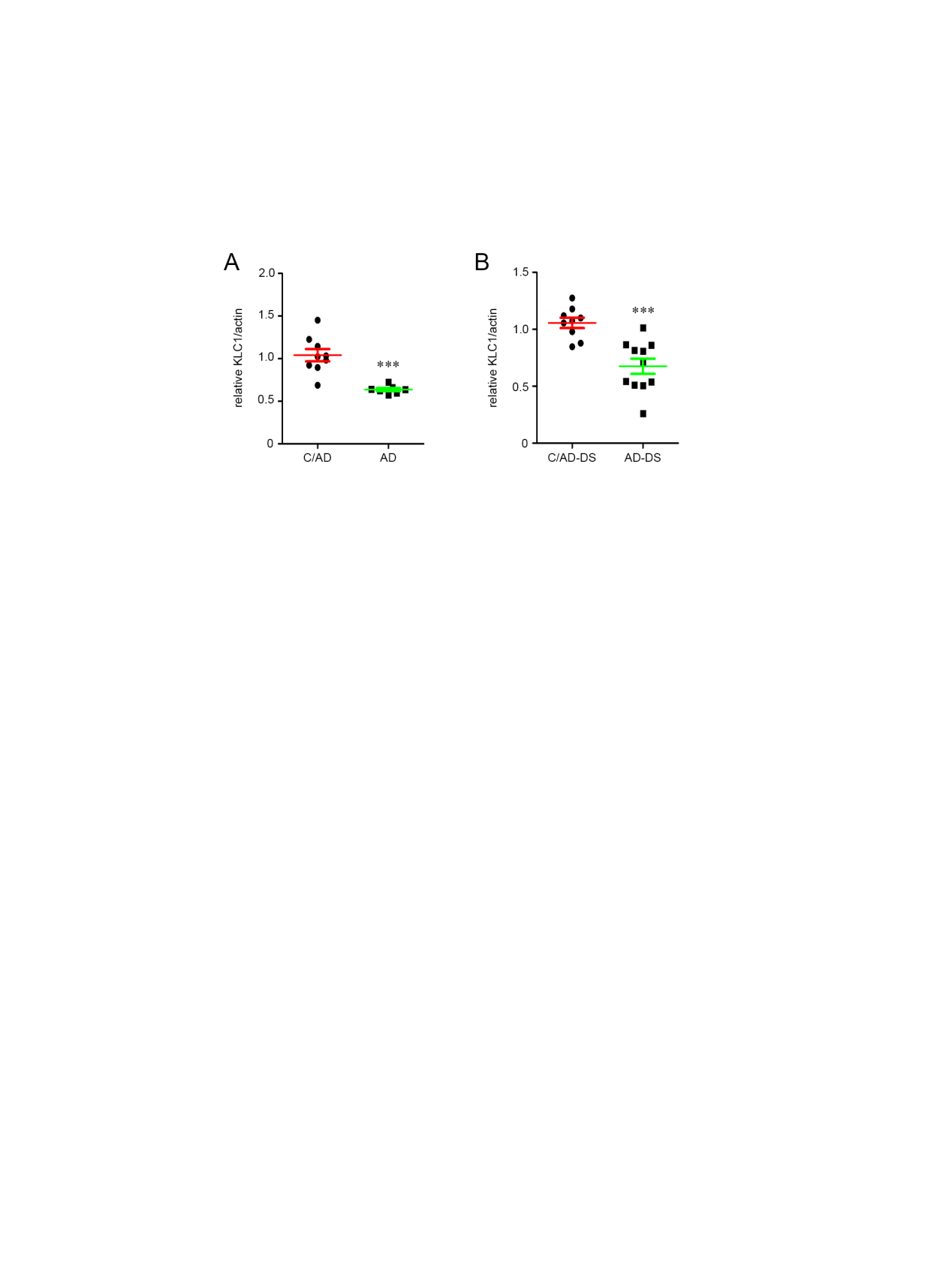


**Supplemental Fig. 4. Reduced KLC1 levels in the frontal cortex of AD and AD-DS examining samples with PMI of ≤ 6 hours.** (A-B) Quantitation and statistical analysis of the levels of KLC1 in the frontal cortex of AD, AD-DS and control samples with PMIs **≤ 6 hours**. n=9 for C/AD, n=7 for AD patients; n=9 for C/AD-DS, n=11 for AD-DS patients. Mann-Whitney test. ***P < 0.001.

**
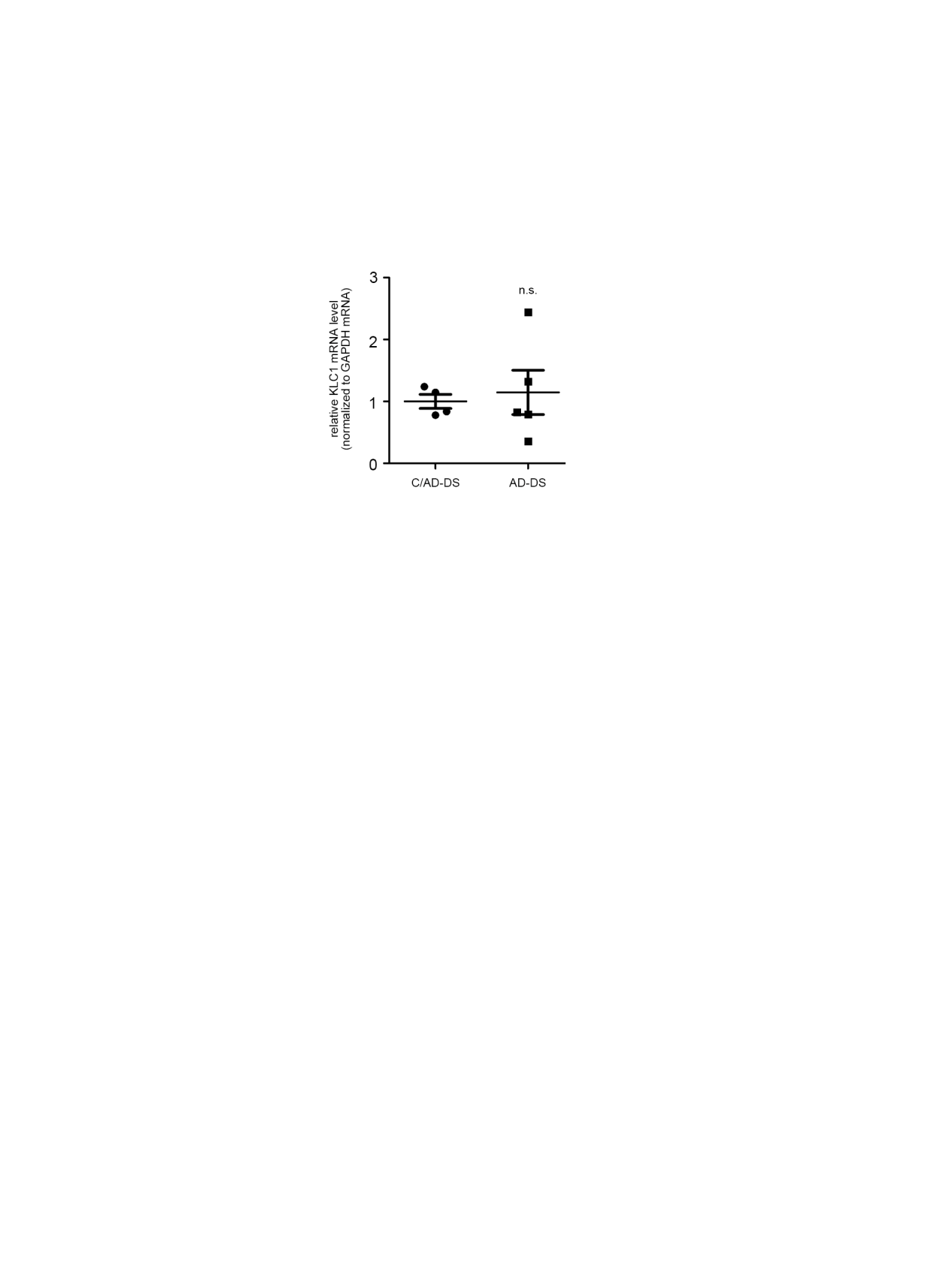
Supplemental Fig. 5. The mRNA levels of KLC1 in the frontal cortex of AD-DS and C/AD-DS brains.** The relative mRNA levels of KLC1 from 9 randomly selected samples were measured by qPCR and normalized to GAPDH mRNA. N=4 for C/AD-DS; n=5 for AD-DS. n.s. non-significant.

**Supplemental Table 1** Spearman’s correlation analysis between KIF5 members when normalized to GAPDH in human temporal cortex and cerebellum samples.

| Sample type |  |  | Sample size (n) | Correlation coefficient (r) | Significance (P) |
| --- | --- | --- | --- | --- | --- |
| C/AD and AD-temporal | KIF5A | KIF5B | 15 | 0.5643 | 0.0284* |
| C/AD and AD-temporal | KIF5A | KIF5C | 15 | 0.8357 | 0.0001*** |
| C/AD and AD-temporal | KIF5B | KIF5C | 15 | 0.6964 | 0.0039** |
| AD-temporal | KIF5A | KIF5B | 7 | 0.3929 | 0.3956 |
| AD-temporal | KIF5A | KIF5C | 7 | 0.5357 | 0.2357 |
| AD-temporal | KIF5B | KIF5C | 7 | 0.7857 | 0.0480* |
| C/AD-temporal | KIF5A | KIF5B | 8 | 0.6905 | 0.0694 |
| C/AD-temporal | KIF5A | KIF5C | 8 | 1.0000 | <0.0001*** |
| C/AD-temporal | KIF5B | KIF5C | 8 | 0.6905 | 0.0694 |
| C/AD and AD-cerebellum | KIF5A | KIF5B | 15 | 0.8929 | <0.0001*** |
| C/AD and AD-cerebellum | KIF5A | KIF5C | 15 | 0.8250 | 0.0002*** |
| C/AD and AD-cerebellum | KIF5B | KIF5C | 15 | 0.8929 | <0.0001*** |
| AD-cerebellum | KIF5A | KIF5B | 7 | 0.8571 | 0.0238* |
| AD-cerebellum | KIF5A | KIF5C | 7 | 0.8571 | 0.0238* |
| AD-cerebellum | KIF5B | KIF5C | 7 | 0.7857 | 0.0480* |
| C/AD-cerebellum | KIF5A | KIF5B | 8 | 0.9048 | 0.0046** |
| C/AD-cerebellum | KIF5A | KIF5C | 8 | 0.9048 | 0.0046** |
| C/AD-cerebellum | KIF5B | KIF5C | 8 | 0.9524 | 0.0011** |

**Supplemental Table 2** Demographics, PMI and clinical diagnosis for AD-DS and C/AD-DS frontal cortex samples used for KIF5 mRNA level analysis.

| Patient | Gender | Age (yrs) | Post-mortem interval (hrs) | Diagnosis |
| --- | --- | --- | --- | --- |
| C/AD-DS 1 | Female | 49 | 7 | Cognitively normal |
| C/AD-DS 2 | Male | 59 | 16 | Cognitively normal |
| C/AD-DS 3 | Female | 54 | 6 | Cognitively normal |
| C/AD-DS 4 | Male | 48 | 6 | Cognitively normal |
| AD-DS 1 | Male | 56 | 16 | Trisomy 21 with AD |
| AD-DS 2 | Male | 57 | 5 | Trisomy 21 with AD |
| AD-DS 3 | Male | 55 | 12 | Trisomy 21 with AD |
| AD-DS 4 | Male | 53 | 24 | Trisomy 21 with AD |
| AD-DS 5 | Male | 65 | 10 | Trisomy 21 with AD |

**Supplemental Table 3A** Spearman’s correlation analysis between KIF5 members and fl-hAPP when normalized to β-actin in human AD frontal cortex samples.

| Sample type |  |  | Sample size (n) | Correlation coefficient (r) | Significance (P) |
| --- | --- | --- | --- | --- | --- |
| C/AD and AD | KIF5A | fl-hAPP | 24 | 0.5096 | 0.0110* |
| C/AD and AD | KIF5B | fl-hAPP | 24 | 0.4148 | 0.0439* |
| C/AD and AD | KIF5C | fl-hAPP | 24 | 0.3443 | 0.0994 |
| AD | KIF5A | fl-hAPP | 12 | 0.5455 | 0.0666 |
| AD | KIF5B | fl-hAPP | 12 | 0.1678 | 0.6021 |
| AD | KIF5C | fl-hAPP | 12 | 0.5105 | 0.0899 |
| C/COMB | KIF5A | fl-hAPP | 21 | 0.5571 | 0.0087** |
| C/COMB | KIF5B | fl-hAPP | 21 | 0.5013 | 0.0206* |
| C/COMB | KIF5C | fl-hAPP | 21 | 0.5429 | 0.0110* |

**Supplemental Table 3B** Spearman’s correlation analysis between KIF5 members and fl-hAPP when normalized to GAPDH in human AD frontal cortex samples.

| Sample type |  |  | Sample size (n) | Correlation coefficient (r) | Significance (P) |
| --- | --- | --- | --- | --- | --- |
| C/AD and AD | KIF5A | fl-hAPP | 24 | 0.5661 | 0.0039** |
| C/AD and AD | KIF5B | fl-hAPP | 24 | 0.6774 | 0.0003*** |
| C/AD and AD | KIF5C | fl-hAPP | 24 | 0.5983 | 0.0020** |
| AD | KIF5A | fl-hAPP | 12 | 0.8322 | 0.0008*** |
| AD | KIF5B | fl-hAPP | 12 | 0.6923 | 0.0126* |
| AD | KIF5C | fl-hAPP | 12 | 0.7692 | 0.0034** |
| C/COMB | KIF5A | fl-hAPP | 21 | 0.4468 | 0.0423* |
| C/COMB | KIF5B | fl-hAPP | 21 | 0.2299 | 0.3161 |
| C/COMB | KIF5C | fl-hAPP | 21 | 0.4000 | 0.0724 |

**Supplemental Table 4A** Spearman’s correlation analysis between KIF5 members and fl-hAPP when normalized to β-actin in human AD-DS frontal cortex samples.

| Sample type |  |  | Sample size (n) | Correlation coefficient (r) | Significance (P) |
| --- | --- | --- | --- | --- | --- |
| C/AD-DS and AD-DS | KIF5A | fl-hAPP | 40 | 0.2415 | 0.1333 |
| C/AD-DS and AD-DS | KIF5B | fl-hAPP | 40 | 0.3038 | 0.0567 |
| C/AD-DS and AD-DS | KIF5C | fl-hAPP | 40 | 0.2897 | 0.0698 |
| AD-DS | KIF5A | fl-hAPP | 21 | 0.7299 | 0.0002*** |
| AD-DS | KIF5B | fl-hAPP | 21 | 0.5584 | 0.0085** |
| AD-DS | KIF5C | fl-hAPP | 21 | 0.7104 | 0.0003*** |

**Supplemental Table 4B** Spearman’s correlation analysis between KIF5 members and fl-hAPP when normalized to GAPDH in human AD-DS frontal cortex samples.

| Sample type |  |  | Sample size (n) | Correlation coefficient (r) | Significance (P) |
| --- | --- | --- | --- | --- | --- |
| C/AD-DS and AD-DS | KIF5A | fl-hAPP | 40 | 0.2739 | 0.0872 |
| C/AD-DS and AD-DS | KIF5B | fl-hAPP | 40 | 0.2801 | 0.0800 |
| C/AD-DS and AD-DS | KIF5C | fl-hAPP | 40 | 0.2433 | 0.1303 |
| AD-DS | KIF5A | fl-hAPP | 21 | 0.6455 | 0.0016** |
| AD-DS | KIF5B | fl-hAPP | 21 | 0.7429 | 0.0001*** |
| AD-DS | KIF5C | fl-hAPP | 21 | 0.6818 | 0.0007*** |

**Supplemental Table 5** Spearman’s correlation analysis between fl-mAPP and KIF5 members in 2N and Dp16 mice using β-actin as a loading control.

|  |  | KIF5 member | Sample size (n) | Correlation coefficient (r) | Significance (P) |
| --- | --- | --- | --- | --- | --- |
| 2N | fl-mAPP | KIF5A | 7 | 0.1071 | 0.8192 |
|  |  | KIF5B | 7 | 0.2857 | 0.5345 |
|  |  | KIF5C | 7 | 0.5000 | 0.2532 |
| Dp16 | fl-mAPP | KIF5A | 7 | 0.3214 | 0.4821 |
|  |  | KIF5B | 7 | -0.1786 | 0.7017 |
|  |  | KIF5C | 7 | -0.1786 | 0.7017 |

**Supplemental Table 6** Spearman’s correlation analysis between PMI and KIF5 members or KLC1 in human frontal cortex samples using β-actin as loading control.

| Sample type |  |  | Sample size (n) | Correlation coefficient (r) | Significance (P) |
| --- | --- | --- | --- | --- | --- |
| C/COMB | PMI | KIF5A | 21 | -0.02409 | 0.9175 |
|  |  | KIF5B | 21 | -0.1771 | 0.4425 |
|  |  | KIF5C | 21 | -0.1029 | 0.6573 |
|  |  | KLC1 | 21 | -0.2070 | 0.3679 |
| AD | PMI | KIF5A | 12 | -0.4545 | 0.1377 |
|  |  | KIF5B | 12 | -0.02797 | 0.9312 |
|  |  | KIF5C | 12 | -0.4266 | 0.1667 |
|  |  | KLC1 | 12 | -0.9231 | <0.0001*** |
| AD-DS | PMI | KIF5A | 21 | -0.2027 | 0.3783 |
|  |  | KIF5B | 21 | -0.3241 | 0.1517 |
|  |  | KIF5C | 21 | 0.005846 | 0.9799 |
|  |  | KLC1 | 21 | -0.4521 | 0.0396* |

**Supplemental Table 7A** Spearman’s correlation analysis between KLC1 and KIF5 members or fl-hAPP when normalized to β-actin in human and mouse samples.

| Sample type |  |  | Sample size (n) | Correlation coefficient (r) | Significance (P) |
| --- | --- | --- | --- | --- | --- |
| C/COMB | KLC1 | KIF5A | 21 | 0.3481 | 0.1221 |
|  |  | KIF5B | 21 | 0.5221 | 0.0152* |
|  |  | KIF5C | 21 | 0.1688 | 0.4644 |
|  |  | fl-hAPP | 21 | 0.5377 | 0.0119* |
| AD | KLC1 | KIF5A | 12 | 0.4895 | 0.1063 |
|  |  | KIF5B | 12 | 0.08392 | 0.7954 |
|  |  | KIF5C | 12 | 0.4056 | 0.1908 |
|  |  | fl-hAPP | 12 | 0.7483 | 0.0051** |
| AD-DS | KLC1 | KIF5A | 21 | 0.04286 | 0.8537 |
|  |  | KIF5B | 21 | -0.01948 | 0.9332 |
|  |  | KIF5C | 21 | 0.08831 | 0.7035 |
|  |  | fl-hAPP | 21 | -0.01818 | 0.9377 |
| 2N | KLC1 | KIF5A | 7 | 0 | 1.0000 |
|  |  | KIF5B | 7 | 0.3214 | 0.4821 |
|  |  | KIF5C | 7 | -0.3571 | 0.4316 |
|  |  | fl-mAPP | 7 | 0.7857 | 0.0362* |
| Dp16 | KLC1 | KIF5A | 7 | 0 | 1.0000 |
|  |  | KIF5B | 7 | 0.6429 | 0.1194 |
|  |  | KIF5C | 7 | 0.6071 | 0.1482 |
|  |  | fl-mAPP | 7 | 0.07143 | 0.8790 |

**Supplemental Table 7B** Spearman’s correlation analysis between KLC1 and KIF5 members or fl-hAPP when normalized to GAPDH in human samples.

| Sample type |  |  | Sample size (n) | Correlation coefficient (r) | Significance (P) |
| --- | --- | --- | --- | --- | --- |
| C/COMB | KLC1 | KIF5A | 21 | 0.3351 | 0.1376 |
|  |  | KIF5B | 21 | 0.2623 | 0.2506 |
|  |  | KIF5C | 21 | -0.02857 | 0.9022 |
|  |  | fl-hAPP | 21 | 0.5076 | 0.0188* |
| AD | KLC1 | KIF5A | 12 | 0.4196 | 0.1745 |
|  |  | KIF5B | 12 | 0.3357 | 0.2861 |
|  |  | KIF5C | 12 | 0.2867 | 0.3663 |
|  |  | fl-hAPP | 12 | 0.2028 | 0.5273 |
| AD-DS | KLC1 | KIF5A | 21 | 0.1234 | 0.5942 |
|  |  | KIF5B | 21 | 0.3247 | 0.1510 |
|  |  | KIF5C | 21 | 0.3182 | 0.1598 |
|  |  | fl-hAPP | 21 | 0.3390 | 0.1328 |

**Supplemental Table 8** Sample sizes and PMIs in referenced papers and this study.

|  | Sample size for control | Sample size for AD/AD-DS | PMI for normal (hrs) (Mean ± SEM) | PMI for AD/AD-DS (hrs) (Mean ± SEM) |
| --- | --- | --- | --- | --- |
| Hares et al., [[42](#_ENREF_42)] | 46 | 49 (AD) | 43 ± 6 | 40 ± 3 (AD) |
| Hares et al., [[48](#_ENREF_48)] | 39 | 47 (AD) | 34 ± 3 | 37 ± 3 (AD) |
| Wang et al., [[43](#_ENREF_43)] | 5 | 4 (AD) | 18.2 ± 2.2 | 21 ± 2.5 (AD) |
| This study | 12 | 12 (AD) | 5.7 ± 1.1 | 7.8 ± 2.1 (AD) |
| This study | 19 | 21 (AD-DS) | 8.8 ± 1.4 | 8.3 ± 1.4 (AD-DS) |

**Supplemental Table** **9** Spearman correlation analysis when two proteins were normalized to β-actin and GAPDH differently in the frontal cortex samples.

| Sample type |  | P value |
| --- | --- | --- |
| C/AD and AD | KIF5A/β-actin and KIF5B/GAPDH | 0.0020** |
|  | KIF5A/β-actin and KIF5C/GAPDH | <0.0001*** |
|  | KIF5C/β-actin and KIF5B/GAPDH | 0.0016** |
| C/AD-DS and AD-DS | KIF5A/β-actin and KIF5B/GAPDH | 0.0258* |
|  | KIF5A/β-actin and KIF5C/GAPDH | 0.0002*** |
|  | KIF5C/β-actin and KIF5B/GAPDH | 0.1600 |
| C/COMB | KIF5A/β-actin and KIF5B/GAPDH | 0.1459 |
|  | KIF5A/β-actin and KIF5C/GAPDH | 0.0059** |
|  | KIF5C/β-actin and KIF5B/GAPDH | 0.0550 |
| AD | KIF5A/β-actin and KIF5B/GAPDH | 0.0446* |
|  | KIF5A/β-actin and KIF5C/GAPDH | 0.0030** |
|  | KIF5C/β-actin and KIF5B/GAPDH | 0.0625 |
| AD-DS | KIF5A/β-actin and KIF5B/GAPDH | 0.0025** |
|  | KIF5A/β-actin and KIF5C/GAPDH | 0.0003*** |
|  | KIF5C/β-actin and KIF5B/GAPDH | 0.0015** |
| C/AD and AD | KIF5A/GAPDH and fl-APP/β-actin | 0.0054** |
|  | KIF5B/GAPDH and fl-APP/β-actin | 0.0010*** |
|  | KIF5C/GAPDH and fl-APP/β-actin | 0.0014** |
| C/COMB | KIF5A/GAPDH and fl-APP/β-actin | 0.0138* |
|  | KIF5B/GAPDH and fl-APP/β-actin | 0.1527 |
|  | KIF5C/GAPDH and fl-APP/β-actin | 0.0256* |
| AD | KIF5A/GAPDH and fl-APP/β-actin | 0.0074** |
|  | KIF5B/GAPDH and fl-APP/β-actin | 0.0446* |
|  | KIF5C/GAPDH and fl-APP/β-actin | 0.0074** |
| AD-DS | KIF5A/GAPDH and fl-APP/β-actin | 0.0124* |
|  | KIF5B/GAPDH and fl-APP/β-actin | 0.2203 |
|  | KIF5C/GAPDH and fl-APP/β-actin | 0.0149* |
| C/COMB | fl-APP/GAPDH and KLC1/β-actin | 0.0894 |
| AD | fl-APP/GAPDH and KLC1/β-actin | 0.0126* |
| AD-DS | fl-APP/GAPDH and KLC1/β-actin | 0.3600 |
